# The importance of genotype-by-age interactions for the development of repeatable behavior and correlated behaviors over lifetime

**DOI:** 10.1186/1742-9994-12-S1-S2

**Published:** 2015-08-24

**Authors:** Jon E Brommer, Barbara Class

**Affiliations:** 1 Department of Biology, University Hill, 20014 University of Turku, Turku, Finland

**Keywords:** Evolutionary quantitative genetics, G matrix, senescence, behavior, animal personality, behavioral syndrome, heritability

## Abstract

Behaviors are highly plastic and one aspect of this plasticity is behavioral changes over age. The presence of age-related plasticity in behavior opens up the possibility of between-individual variation in age-related plasticity (Individual-Age interaction, IxA) and genotype-age interaction (GxA). We outline the available approaches for quantifying GxA. We underline that knowledge of GxA for behaviors is an important step in reaching and understanding of the evolution of plasticity in behavior over lifetime. In particular, the heritability (repeatability) and/or the rank order of behavior across individuals are predicted to change across ages in presence of GxA. We draw on the theory of reaction norms to illustrate that GxA, when present, is likely to lead to developmental changes in the magnitude and possibly sign of the genetic correlation between behaviors (behavioral syndrome). We present an overview of the literature on changes in the ranking of individuals’ behavior across ages, and in the correlation between behaviors. Although all studies were carried out on the phenotypic level, they overall suggest clear scope for increased study of GxA as a process explaining age-related plasticity in behaviors. Lastly, we throughout emphasize that many of the approaches and underlying theory of GxA is applicable to the study of IxA, which is informative as it presents the upper limit of GxA, but is also a more attainable target of study in many systems. Empirical work aimed at understanding IxA and GxA in behavior is needed in order to understand whether patterns predicted by theory on plasticity indeed occur for age-related plasticity of behavior.

## Introduction

**Fig. 1:**
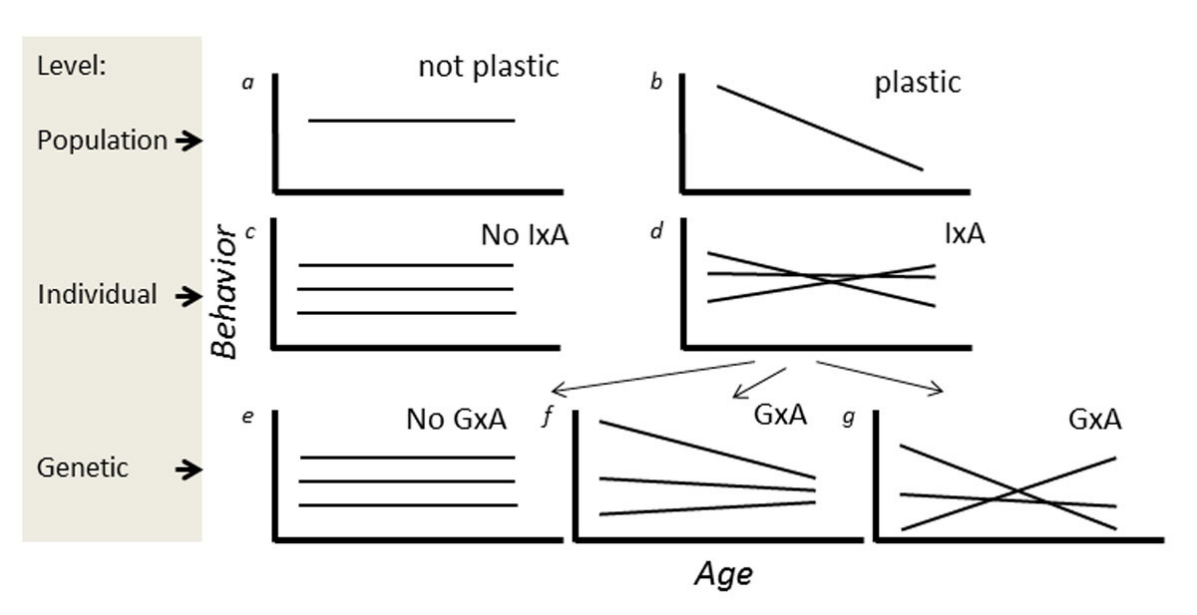
Schematic illustration showing how plasticity in behavior with age can occur on different levels. For simplicity, only linear plasticity between behavior and age are drawn here, but the same hierarchical structure applies to non-linear relationships. On the population level, (a) the age-specific mean behavior may be invariant with age, but (b) may also vary across ages. On the individual and genetic level, deviations from these age-specific means are considered. (c) All individuals show the same deviation from the average behavior at all ages, and there hence is no between-individual variation in plasticity over ages; no Individual – Age interaction (IxA). Alternatively, (d) individuals differ in their age-specific deviation from the age-specific means, thus showing variation in plasticity (IxA). Despite the presence of IxA, (e) Genotype – Age interaction (GxA) may be absent, or (f) GxA occurs without the ranking of genotypes changing across ages (reaction norms not crossing within the range of ages considered), or (g) GxA where the ranking of genotypes changes (reaction norms cross).

Behavior is often systematically affected by environmental conditions and by internal states. Such gradients driving plasticity may be an external environmental variable (e.g. temperature), but can also be the age of the organism[1]. In this paper, our focus is explicitly on plasticity in behavior in response to the age at which the behavior is expressed. Plasticity of behavior across ages can be viewed on the level of the population (mean behavior varies over ages), the individual (behavior of the same individual varies over ages) and the genotype (Fig. 1). For illustration purposes, plasticity is in this figure and throughout this paper presented as linear (gradual) changes in the expression of a behavior over ages. Linearity depicts the simplest form of plasticity, but clearly plasticity can have a more complicated (non-linear) shape, and the approaches and concepts applied here apply also to non-linear plasticity. Importantly, whenever a trait is plastic, we may expect variation in the degree of plasticity across individuals (in the context of age-specific expression, Individual – Age interaction; IxA, Fig. 1, cf.[2]). IxA signals that some individuals show a greater or smaller degree of plasticity in behavior when ageing than others. For example, an individual shows low plasticity when it is approximately equally aggressive when assayed at different ages, whereas other individuals may be highly plastic, being e.g. very aggressive when young but expressing much lower levels of aggression when old. One part of the variation between individuals in their plasticity may be on the additive genetic level, which is termed Genotype – Age interaction (GxA, Fig. 1). Below we provide more details on quantitative genetic concepts and approaches for estimation of IxA and GxA (see also the glossary Additional file 1), but we first outline three reasons to be interested in between-individual (IxA) or between-genotype (GxA) variation in the plasticity of a behavior over ages.

Firstly, a range of students of behavior ask whether variation underlying between-individual differences measured at a certain age is representative for differences between these individuals at other ages. The (implicit or explicit) assumption in most studies is that a behavior shows constant between-individual or additive genetic variance over all age classes and that the ranking of trait values (behavior expressed by an individual) does not change across age[3,4]. Consider, for example, aggressive behavior. The study of IxA and GxA in aggression would answer questions as: Is the individual that is the most aggressive juvenile also expected to be the most aggressive adult or does the ranking of individuals in terms of their aggressive behavior change as they age? Does the repeatability and heritability of aggression change during the lifetime of individuals, and, if so, does it increase or decrease? Essentially, knowledge of the presence and the extent of variation in plasticity between individuals and genotypes in a behavior allows answering these questions (cf. [5]). These answers have relevance in a variety of fields. For example, animal breeders are interested in the above listed questions when they want to develop behavioral assays which can be conducted in young animals and predict the behavior of the individual at a later age (e.g. [6]). Researchers of human behavior are interested in understanding at what age pronounced differences between individuals arise (e.g. [7]) as well as whether behavior of individuals in one age group predict their behavior in another [8]. Behavioral ecologists study the above questions, because between-individual differences in behavior is considered the hallmark of animal personality, a current focus in behavioral ecology [8,4].

Secondly, the study of GxA relates to fundamental aspects of evolutionary biology, because evolutionary theories of senescence [9,10] predict the presence of GxA in traits showing senescence [11-13]. Senescence can be defined as a decline in organismal performance with age, and senescence occurs in practically all taxa [14,15]. Senescence in, say, aggression could cause individuals to become less aggressive with age, i.e. to show age-related plasticity. Evolutionary theories explaining why senescence occurs, hinge on the assumption that the trait which shows a senescent decline is related to an individual's fitness. Meta-analysis shows that many behaviors have selective consequences [16,17]. Thus, some behaviors are a measure of organismal performance and an explicit investigation of plasticity over lifetime in such fitness-related traits from the perspective of senescence would hence be of interest. One would expect an age-related change in behavior towards values with lower fitness on the population level (i.e. senescence). Whenever a behavior shows such a senescent decline, the study of between-individual and especially between-genotype variation in the rate of senescence (IxA and GxA respectively), provides one approach to test for the “fingerprint” of evolved senescence [12,13].

Third, knowledge of GxA in behavior is of potential interest for those who aim to gain insight in the causes of variation in behavior on the proximate level in terms of either identifying genetic loci associated with variation in behavior or studying the physiological mechanisms underlying behavior. Behaviors without GxA are indicative of a clear consistency in behavior over ages, and hence signal a behavior where the proximate determinants (genes, physiological mechanisms) are likely to be the same independently of organismal age. In contrast, behaviors with GxA require a more careful dissection because these suggest that genetic associations and physiological mechanisms are acting in an age-dependent manner.

The main aim of this paper is to flag the potential importance and relevance of considering between-individual and between-genotype variation in age-related plasticity in behavior (IxA and GxA respectively). To this end, we start with a short primer on quantitative genetics and then focus in some detail on approaches used in the estimation of GxA as well as the kind of information one obtains on the basis of these approaches. These approaches are established in quantitative genetics, but are applicable also in the estimation of IxA such that they are of interest even when the focus is mostly on understanding between-individual differences rather than genetic effects. We emphasize what the putative existence of GxA would imply for changes in the heritability (and repeatability) of behavior during the lifetime of individuals as well as the correlation in behavior across ages. Because the study of GxA in behavior is largely unexplored to date, we present an overview of the patterns in age-related changes in repeatability assuming it presents an upper limit of the heritability [18], and changes in the ranking of individuals’ behaviors across ages. We then explore the implications of the presence of GxA for the correlation between behaviors when such correlations are measured at different ages. Correlated behaviors are termed behavioral syndromes. Such syndromes are a defining feature of many behaviors [3,19]. Although most behavioral syndromes described to date are based on phenotypic correlations, there is reasonable evidence for genetic correlations underlying syndrome correlations [20]. We outline, based on the norms of reaction framework [21], how the putative presence of GxA in behavior predicts age-related changes in the strength and possibly sign of a behavioral syndrome. We present an overview of the literature exploring changes in the phenotypic correlation between behaviors over age. Based on our overview of approaches and the literature, we end with a perspective for future work on this exciting research question.

## Primer in quantitative genetics

In this section, we briefly summarize some of the core concepts in quantitative genetics. Some of the concepts mentioned in this section are explained in the glossary (Additional file 1 ). The quantification of age-related changes in (genetic) (co)variances requires a quantitative genetic approach [22]. Quantitative genetics provides a statistical description of the relative contribution of different sources of genetic versus non-genetic effects underlying phenotypic variance in a quantitative trait. A quantitative trait is any trait which is not inherited Mendelian, and is typically viewed as a measurable quantity on the continuous scale (e.g. body length). However, a quantitative trait can be meristic (e.g. number of bristles) or discrete (e.g. winged vs. unwinged morphs) assuming there is a latent underlying variable triggering a switch in discrete phenotypes once a certain threshold value is exceeded [23]. Thus, most behaviors are amenable for analysis in the quantitative genetic framework.

Quantitative genetics builds on the assumption that variation in quantitative traits is caused by many loci of small effect, an assumption typically confirmed by molecular genetic analyses [22,24,25]. The breeding value may be constant over all ages in which the individual is expected to express the same value for the focal behavior at each age. However, when GxA is present, the magnitude and possibly the sign of the effects of loci on the focal trait changes as the individual ages. Some loci which earlier did not have an effect are turned on, others may be turned off, or the same genes increase/decrease in their effect. Thus, the presence of GxA is assumed to signal a change in the genetic underpinning of the behavior.

Quantitative genetics’ standard view is that phenotypic (co)variance is the resultant of a compounding of different, hierarchically structured effects, each generating its own (co)variance. That is, phenotypic variance is partitioned into additive genetic (co)variances and other sources of (co)variances including residual (co)variances. To this end, information on the relatedness between individuals (i.e. a pedigree) is a minimal requirement for partitioning out the additive genetic (co)variances, but further tools may include a breeding design or artificial selection[22]. By marking individuals and their offspring for a number of breeding episodes, one can obtain a multi-generational pedigree. In species where parentage is difficult to assign based on observation, one needs to resort to molecular genetic assignment methods [26]. Pedigrees can be assembled both in wild populations and populations reared under more controlled environmental conditions. Depending on the organism, a number of breeding designs may be applicable to laboratory or (semi-)domestic populations; detailed treatment of these can be found in textbooks (e.g.[18,22]). Cross fostering of offspring is one breeding design with potential to improve quantitative genetic estimates, which is also applicable to many wild, domestic and laboratory populations [27]. While certainly not all organisms are amenable for such approaches, it is clear that many laboratory, domesticated or wild systems which are currently used in the study of behavior would allow construction of a pedigree and hence quantitative genetic analyses.

A general approach for analysis of most of the pedigree or breeding design data described above, is to use a specific form of linear mixed model, where information on the relatedness between individuals is included. This so-called “animal model” uses individual-specific measurements to estimate quantitative genetic parameters and was originally developed by animal breeders, but is increasingly used in various fields [22,28]. A starting point in getting acquainted with this approach, including tutorials, is provided by ref.[29]. The animal model can flexibly include information on various factors which are likely to cause resemblance between individuals, for example the brood in which siblings were reared or the identity of their mother. In this paper, we follow the convention in assuming that variance in phenotypes are due to additive genetic effects and various other, non-heritable (environmental) effects. Thus, non-additive genetic effects (dominance and epistasis) and their interactions are not considered in this paper. Non-additive genetic effects can be incorporated within the animal model framework, but doing so requires much information on specific relatives and is hence challenging to estimate. In general, dominance genetic effects are important, both in the study of senescence [30] and in the study of behavior [17,31]. Because these effects are challenging to estimate, a reasonable first step, nevertheless, is to get a handle on the additive genetic effects, bearing in mind the underlying assumption that non-additive genetic effects are ignored.

## Age-related changes in (co)variances created by IxA and GxA

**Fig. 2:**
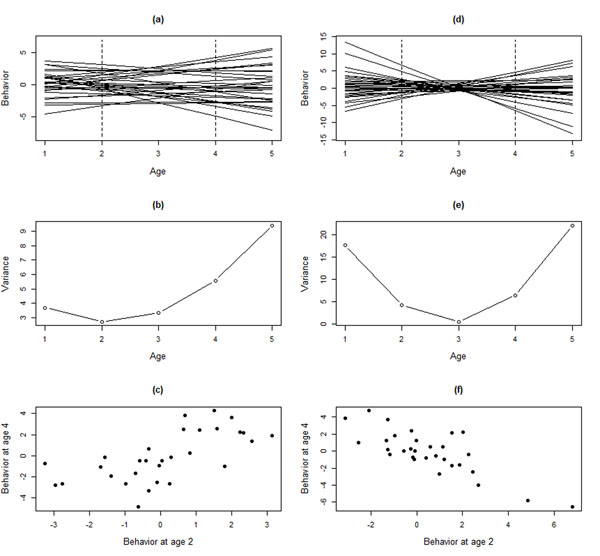
Illustration of how different patterns of GxA lead to different patterns of additive genetic variance in behavior and cross-age correlations. For simplicity, changes in the breeding values with age are depicted as lines, but qualitatively the same patterns may arise under different functional forms. For 30 genotypes, the reaction norm elevation (defined here as the expected behavior at age 3) and the plasticity (slope of how the deviation relative to age-specific mean behavior is expected to change across ages) were randomly drawn from a multivariate normal distribution. Panel (a), (b) and (c) denote the situation where reaction norm elevation and the plasticity are correlated (variance in elevation = 4, variance in slope = 1, r = 0.65), which leads to (a) reaction norms mostly “fanning out”, producing (b) an increase in additive genetic variance with age, and (c) a positive genetic correlation between behaviors at age 2 and 4. Panels (d), (e), and (f) display when reaction norm elevation and slope are not correlated (variance in elevation = 1, variance in slope = 4, r = 0), which leads to (a) crossing reaction norms which (e) lead to a non-linear pattern in additive genetic variance over ages and (f) a negative genetic correlation between behavior at age 2 and 4.

A central prediction of GxA (or IxA) is that, when it is present in the population, the variance between genotypes (or, for IxA, individuals) changes across ages (Fig. 2). The treatment of GxA in this paper is equivalent to general insights related to the presence of Genotype – Environment interactions as discussed in textbooks (e.g [18,22]). An overview of between-individual variation in plasticity and its relation to genetic variation in plasticity is provided by ref. [2]. The concept of between-individual variation in plasticity within the context of studies on behavior is discussed by ref. [32].

GxA (and IxA) can be present in one of two forms. One in which the ranking of the expected behaviors expressed by individuals is maintained across all ages considered (Fig. 2a,b,c), in which case the additive genetic variance increases (decreases) across ages, but correlations in expression differences between genotypes are maintained. In contrast, GxA may also take the form where the ranking changes as the organism ages (Fig. 2d,e,f), in which case there is a minimum in the additive genetic variance at some age. Despite the fact that these two forms of GxA have different biological implications, the only difference between them is whether the crossing of the GxA reaction norms occurs outside the range of ages considered (Fig 2a) or within the range of ages considered (Fig. 2d).

Typically, the study of plasticity of behavior over age, and variation in this plasticity, is based on repeated measures of behavior made on individuals during (part of) their lifetime. It is important to note that although each individual is likely to be a unique genotype, the demonstration of IxA does not constitute proof of underlying GxA. This is because, in broad terms, the expected behavior of an individual at a certain age is not only determined by its genotype, but also by its permanent environment. These permanent-environment effects may cause between-individual differences to have no additive genetic basis. Thus, the presence of plasticity in behavior itself does not inform whether there is variation in plasticity between individuals (IxA), nor does the presence of IxA inform on GxA. Instead, IxA and GxA are properties which need to be estimated explicitly. Provided information on the relatedness across individuals is available, quantitative genetic approaches, which we outline below, can be used to quantify GxA.

## How to estimate GxA

The core interest in modelling IxA and GxA is in the (co)variances among and between age-specific performances, as outlined in the previous section. In this section, we provide a brief overview of possible approaches to study IxA and GxA in behavior with the main objective to clarify what kind of quantities may be estimated. Although the focus in this section is on GxA, the main approach and logic applies similarly to the estimation of IxA, except that in the latter case between-individual (co)variances are to be estimated instead of additive genetic (co)variances. In order to quantify IxA and GxA, one needs to measure a behavior which is repeatedly expressed by the same individual at different ages during its lifetime. Because the interest is in age-related changes, the focal behavior shows plasticity on the individual level (i.e. the same individual may behave differently at different ages). Plasticity on the individual or genetic level (as illustrated schematically by lines in Fig. 1 and 2) concern deviations from the age-specific mean of the individual and breeding value, respectively[22]. Thus, even when the mean behavior shows a non-linear pattern over ages (e.g. aggression increases early in life, but then decreases), the issue is whether the individual-specific deviations and breeding value deviations from these age-specific mean values varies over age classes. Thus, despite IxA and GxA being illustrated here with linear lines (Fig. 1 and 2), the population-level pattern of behavioral plasticity may be more complex (illustrated in additional file 2). Statistically, the focus on deviations from age-specific mean values necessitates correcting age-specific behavior for its age-specific mean (e.g. by inclusion of age as a factorial fixed effect or by considering the behavior at each age, as detailed below).

For estimation of GxA, the basic approach is to recognize that a behavior expressed at a certain* age* is potentially different (in terms of the additive genetic effects underlying it) than at* age*+1 and so on. Thus, the behavior is considered to have, what quantitative geneticists term, different character states [22]. Conceptually, the model structure is multivariate , and follows the same procedure as if the focal trait measured at each character state were separate traits (see e.g. [29] for details on how to implement such models). Under the character-state model, one obtains estimates of

$$G=\left[\begin{array}{cccc} V_{A}^{1} & C_{A}^{2,1} & \cdots \; & C_{A}^{\omega ,1}\\ C_{A}^{1,2} & V_{A}^{2} & \cdots \; & C_{A}^{\omega ,2}\\ \vdots & \vdots & \ddots & \vdots \\ C_{A}^{1,\omega } & C_{A}^{2,\omega } & \cdots \; & V_{A}^{\omega } \end{array}\right],$$

which is the genetic (co)variance matrix denoting in its diagonal the additive genetic variance *V_A_* for the behavior at age classes 1, 2,… ω, where ω is the last age class considered. In the off-diagonal, the pair wise genetic covariances (*C_A_*) are denoted between the age classes. Again, depending on the design of the study, the full multivariate mixed model used to estimate** G **(eq. 1) will include other matrices which may or may not be similarly structured as **G** describing the (co)variances at other levels including the residual level.

The full character-state approach requires estimation of many parameters. The **G** matrix (eq. 1) for age classes requires ω(ω + 1)/2 additive genetic (co)variances to the estimated, which rapidly becomes demanding. For example, three age classes require six, five age classes 15, and seven age classes requires 28 genetic parameters. One possibility to reduce complexity is to group some age classes into *a priori* determined biologically relevant age groups (e.g. juveniles, sub-adults, adults). Another approach is to use a function-valued trait approach to approximate the **G** matrix under simplifying assumptions requiring the estimation of a reduced set of parameters (reviewed by [33]). The main function-valued trait approach used is the so-called infinite-dimensional model [1], which assumes that a set of *n*-orthogonal polynomials can describe the **G** matrix. The variances and covariances in the parameters describing these polynomial functions are typically estimated using random regression [34], and within the animal model framework such a model is known as a Random Regression Animal Model (RRAM; [35,2]. The RRAM is a statistical implementation of what is an intuitive conceptual visualization of GxA, because it concerns the “drawing” of reaction norms of how the breeding value for a behavior depends on the age at which it is expressed (cf. Fig. 1 and 2).

The hierarchical nature of plasticity can be illustrated by considering two general formulations of random regression. The trait *z* for individual *i* as a function of its age *age* is, at the phenotypic level, described by

$$z_{i,age}=\mu +AGE_{F}+f_{ind}\left(x,age\right)+\varepsilon_{i,age},$$

which can be partitioned further into

$$z_{i,age}=\mu +AGE_{F}+f_{a}\left(x,age\right)+f_{pe}\left(x,age\right)+\varepsilon_{i,age},$$

where μ is the overall fixed-effect mean, *AGE_F_* denotes the inclusion of age as a factor in order to model age-specific means. Functions *f_ind_(x, age), f_a_(x, age)* and *f_pe_(x, age)* describe an orthogonal polynomial of order *x* on the level of the individual, additive genetic and permanent environment, respectively. These polynomials capture the deviations from the age-specific fixed effect means. In practice, the coefficients of the polynomial are assumed to stem from a multivariate normal distribution [35]. Lastly *ε_i,age_* is the (age-specific) residual for individual *i*.

The reduction in the number of parameters when using a RRAM to estimate **G** is potentially large. For example, when a second-order polynomial (linear and quadratic reaction norms; *a*_0_ + *a*_1_*× age* + a_2_*× age*^2^) are assumed, the RRAM estimates these coefficients as stemming from the multivariate normal covariance matrix

$$K_{a}=\left[\begin{array}{ccc} V{\text{a}}_{0} & C{\text{a}}_{0,1} & C{\text{a}}_{0,2}\\ C{\text{a}}_{0,1} & V{\text{a}}_{1} & C{\text{a}}_{1,2}\\ C{\text{a}}_{0,2} & C{\text{a}}_{1,2} & V{\text{a}}_{2} \end{array}\right],$$

where *V* and *C* are variance and covariance, respectively, in the breeding value for the intercept (*a*_0_), the linear slope (*a*_1_) and the quadratic slope (*a*_2_) of the reaction norms. For a polynomial of order *x*, **K_a_** will be of dimension *x* × *x*.

Once the random-regression covariance matrices **K_a_** is obtained, it can be transformed to **G** for any ages considered as appropriate character states[36], and confidence intervals can be estimated([37]. Importantly, it is this transformation of the covariance matrix of reaction-norm properties to the character-state (**K_a_**, eq. (3) to **G** (eq. 1); or, when studying IxA, from **K_ind_** to the individual matrix **ID**, [5]) which provides information on the age-specific repeatability and cross-age correlations (changes in ranking across ages). Changes in repeatability and the level of crossing cannot be distilled directly from investigation of the covariance/correlation between reaction-norm elevation and slope (i.e. **K**). This is because even when the reaction norms show considerable crossing (low or negative correlation between elevation and slope), the critical aspect is whether this crossing occurs within the range of ages considered and on the variances in elevation and slope (detailed in [5]).

A RRAM will assume that the additive genetic variances behave as a smooth function over all ages, one order higher than the polynomial of the underlying reaction norms[1]. For example, first-order polynomials assume that the additive genetic variance is a second-order function of age (cf. Fig. 2). Importantly, if the model does not capture the true biological pattern then it risks producing a misleading pattern. Although this statement applies to all statistical models, it is especially true for RRAM where models with higher-order polynomials (i.e. complicated patterns of changes in additive genetic variance over age) may not converge. As a consequence, RRAM runs a clear risk of not being able to fully assess statistically whether the final model is a simplification– the best possible (but incomplete) description– or actually the best possible and approximately correct description of the phenomenon of interest. Thus, RRAM risks producing spurious results regarding what, in the end, is the key focus of the analysis: Does additive genetic variance increase with age? An important aspect of using Random Regression (for modelling GxA or IxA) is hence to critically check the outcome with respect to models based on the character-state approach (where older age classes are combined) as well as the phenotypic variances predicted by the model and found in the data (see [5] for a conceptual illustration). Although random regression is the most common function-valued trait approach, it should also be noted that other function-valued trait models can be used to approximate eq. (1). For example, genetic (co)variances may be assumed to stem from an auto-correlation across ages [38]. A full treatment of the recommendations in fitting function-valued trait models is outside the scope of this paper; overviews are given in ref[12] and [33].

## Phenotypic patterns of behavioral variation over lifetime

In this section, we explore the literature on age-related behavioral plasticity to explore central predictions of GxA. Because there are few studies which have explicitly quantified GxA, we here focus on

(1) changes in the repeatability of behavior over ages, assumed to be indicative of changes in between-individual variance over ages. Repeatability presents the upper limit of heritability [18]. Assuming residual variances are approximately constant over ages, absence of a change in repeatability over ages suggests, although it does not prove, an absence of GxA.

(2) We explore age-related changes in the ranking of behavior, which have been estimated primarily as the phenotypic correlation of a behavior expressed by individuals at different ages. Under the assumption that the behavior is heritable, and that age-specific residuals are not correlated, such phenotypic correlations should present the lower expectation of the genetic correlation in behavior between ages (cf. [39]).

We performed a search in ISI Web of Knowledge using different combinations of keywords (“personality”, “temperament”, “stability”, “consistency”, “repeatability”, “development”, “age”) and selected only studies on behavior reporting estimates of repeatability for, or rank-order consistency between consecutive ages or ontogenetic stages. However, most of these studies did not test the statistical difference between ages and only provided information on whether each point estimate differed significantly from 0 (p<0.05). In total, we found 39 publications [6,40-77], summarized in additional file 3. Below we summarize the findings of these studies.

## Changes in the repeatability of behavior over lifetime

The importance of quantifying changes in the repeatability of behavior with age has been recognized (e.g. [78]). Behavioral ecologists, in particular, emphasize that the direction of change in repeatability over age is difficult to predict. On one hand, repeatability can be expected to be lower in juveniles because of ongoing developmental changes. On the other hand, repeatability can be higher in juveniles because developmental trajectories are highly constrained[78]. Nevertheless, individual differences in behavior are typically assumed to remain consistent over the individual's lifetime. The average repeatability of behaviors is 0.37 (reviewed by [79]). However, most of the behavioral tests have been conducted in one ontogenetic stage (adults) and repeatability decreases with the interval between measurements [79]. This decrease suggests that repeatability in behaviors, which is not very high, can be even lower across life-stages.

Few studies specifically addressed the question of lifelong stability of repeatable behavioral differences in animals; repeatability of behavior at old age is typically not explored. For example, animal breeding research benefits from the largest body of literature on the development of personality, as the interest of breeders is to predict the behavior of animals or other traits of interest, based on behavior expressed early in life. However, many of these studies are restricted to a relatively short time-span (from birth to maturity) and do not explore the question of aging, which is of interest mainly for behavioral ecologists and psychologists. The same observation can be made in behavioral ecology where most studies on the development of behavior focus on the earliest life-stages, rather than the entire lifespan. Changes in repeatability over age have been investigated in a meta-analysis [79] where no significant difference was found among age groups. However, most of these studies include only behavior measured within one ontogenetic stage. In the few studies reporting repeatability of behavior across ontogenetic stages, repeatability is on average lower in juveniles than in adults (0.50 for juveniles 0.58 for adults, 9 traits in total) but this difference is small and concerns only vertebrates[40,41,80-82]. Recently, a mixed-model approach (an individual-level version of the random regression model outlined above) was used to arrive at changes in the repeatability of crayfish boldness (0.19, 0.25, 0.44 at day 1, 36 and 72, respectively) and latency to feed (0.50 and 0.47 at day 1 and 68 respectively)[42]. On the other hand, other studies reported a higher behavioral consistency (rank-order correlation between consecutive tests) in juveniles than in adults [83,84].

## Changes in the correlation of a behavior between age classes

**Fig 3:**
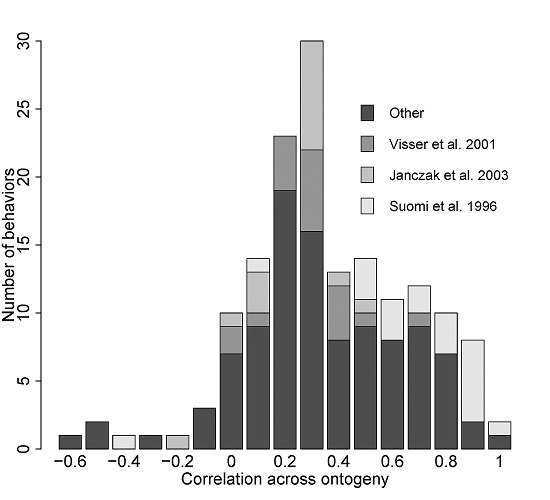
Distribution of the rank-order correlations of behavioral traits across ontogeny. Studies reporting more than 8 traits are represented in different colors than the other studies. Studies not reporting their estimates of non-significant correlations are not included. Studies are listed in additional file 3.

The correlation in behavior across age classes is regularly measured in studies on animal behavior. Typically, behavior is measured once at different ages and a (Spearman rank) correlation is calculated for the behavior between consecutive ages (reported in additional file 3 as Cor age-age+1). These correlations indicate how the rank order of measurements (i.e. phenotypes) for behavioral traits changes, as individuals age. Three studies [55-57] only reported significant correlations, because the objective in these studies was to arrive at methods for behavioral assessment of adult (2.5 year old) horses on the basis of their behavior as a fawn. Thus, these latter estimates inform us of the upper expected value of a correlation between age classes, which is around 0.6 for behavior in horses. When the results of all studies which have reported both significant and non-significant correlations are taken together, they suggest that behavioral traits are moderately correlated across ontogeny: The modal correlation between the first and second age class (Cor1-2 in additional file 3) is 0.3 (Fig.3). Although three studies contributed a disproportional amount of estimates, a similar pattern is also visible in the other studies (Fig. 3). Furthermore, we note that cross-ontogeny correlations can reach very high values for certain traits and shorter inter-test intervals (see e.g.[45]). Low cross-age phenotypic correlations are consistent with the pattern of rank-changes in behavior predicted when GxA occurs such that the reaction norms cross within the range of ages considered (cf. Fig. 1d). Contrary to repeatability estimates, cross-ontogeny correlations can be negative which means that the rank order of individuals for one trait will be reversed across ontogeny, as predicted when the reaction norms cross within the age interval considered. Overall, there is little evidence for such changes in rank in the studies included in our literature overview. Negative cross-age correlations occur in only a few cases (22/467 correlations) and in most of these cases (17/22 correlations), the point estimates in fact did not differ significantly from 0. Nevertheless, our overview underlines that among studies measuring behavior at more than two ages, the correlations between consecutive ages can clearly change as the organism ages (Additional file 3), such that behavior of an individual at early age is not necessarily a good predictor of its behavior at late ages. Nevertheless, none of these studies has tested the statistical difference between rank-order correlations at different ages as the age-related change in ranking was not a specific focus of these studies.

The development of personality has been extensively studied in humans, not only from childhood to maturity but also in old ages, to understand how personality changes with age due to genetic or environmental factors and to be able to predict and prevent maladaptive changes in personality. Most psychology studies agree that personality is characterized by continuity and change [85-88]. On one hand, there is empirical evidence that rank-order consistency in personality traits is moderate from childhood to adulthood and increases with age to reach high values in late adulthood [88]. On the other hand, personality traits do not become fixed in adulthood and are still likely to undergo some changes at the population mean level but also on the individual level, as a result of the interaction of the individual and his or her environment. Indeed, some studies have found significant individual differences in age-related changes in personality traits [89-92]. Moreover, rank-order consistency has been found to increase with age and decrease with the time interval between tests [88]. More recently, human personality research has started to include individuals older than 60 years and found a curvilinear pattern for rank-order consistency with age which, after reaching a maximum at around 50 years, started to decrease [7,93-96]. These findings imply that the rank orders of individuals are more and more likely to be maintained from childhood to mid-adulthood when the individuals grow old, but this consistency decreases after a certain age. Again, rank-order changing GxA may be underlying such patterns.

The importance of genetic vs. environmental determination of personality and its development has been long studied in psychology. Firstly, common patterns of change in the 5 human personality dimensions over age and across cultures have been found even in older ages, showing that genes play an essential role in personality development across the entire lifetime [97]. Secondly, heritability of personality dimensions has been estimated at different ages using long-term studies of monozygotic and dizygotic twins. Heritability seems to be relatively consistent in early life [98,99] but there is evidence for an increase during adolescence and young-adulthood [100,101] and a decrease in late adulthood [102,103]. Indeed, the rank-order changes across ages have been attributed to both genetic and environmental causes, genetic factors being the main contributor to inter-individual differences in behavior in early life whereas environmental effects accumulate over the lifetime to become the main contributor in older ages [102,103]. This is because the genetic variance is relatively stable over the ontogeny while the environmental variance increases with age. As a result, heritability is expected to decrease after a certain age. Hence, studies of human behavior have specifically addressed the question of personality development over the entire lifetime, while separating genetic and environmental factors with evidence that GxA acts as a potential cause of individual differences in age-related changes in personality.

Taken together, the overview of literature of behavioral studies in this section suggests there is considerable scope for changes in the ranking of individual expression of behavior during development. In particular, the modal phenotypic correlation of a behavior in animals across ages was 0.3 (Fig.3). Although this is a positive correlation, it suggests that many behaviors are not particularly well correlated from one age class to the next; variation in behavior in one age class tends to predict 0.3^2^ = 9% of variation in behavior in the next age class (where many age classes are arbitrarily defined and often concern a short time span). However, studies done thus far are largely restricted to the phenotypic level and do not separate correlations due to additive effect of genes from residual correlations or permanent environmental correlations. Hence, changes in the rank order of individuals for certain behavioral traits across their lifetime do not necessarily indicate the presence of GxA. Indeed, phenotypic correlations typically underestimate the magnitude of genetic correlations, although their sign is generally consistent with the sign of genetic correlations [104]. Thus, the genetic correlation of behavior across ontogeny is likely to, on average, be greater than 0.3, but answering this question for any particular study can only be done by explicitly calculating genetic correlations across ontogeny.

## Correlation between multiple traits across ages: theoretical considerations

Different behaviors are often correlated. In the behavioral ecology literature, such correlational structures are termed behavioral syndromes[3]. Some authors argue that behavioral syndrome correlations should be quantified as between-individual correlations, requiring a partitioning of (co)variances in between-individual and residual (co)variances[105]. Supporting this view is the notion that the between-individual covariance/correlation focuses on the same hierarchical level of interest as repeatability, which is a measure of the between-individual variance[105]. However, the majority of studies document behavioral syndromes on the phenotypic level only. Although there are studies quantifying the genetic correlation between two or more behaviors (reviewed in [104]), there are few studies which have estimated the genetic correlation between behaviors at multiple ages. In this section, we first illustrate whether and how the genetic correlation between traits is expected to vary over ages, given the presence of GxA. We then present an overview of the literature on age-specific phenotypic correlations between behaviors in order to explore whether patterns predicted to exist under the presence of GxA are found on the phenotypic level.

**Fig 4:**
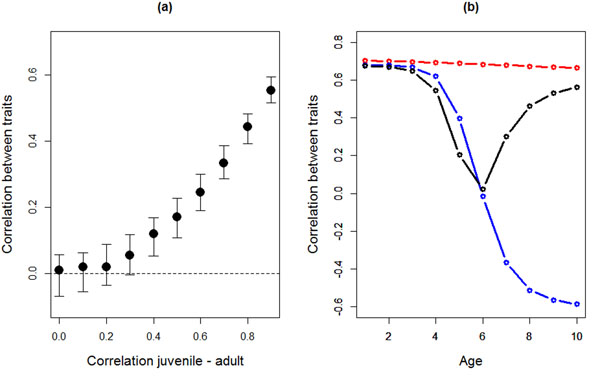
Expected correlation between two behaviors over various ages depending on the form of GxA. (a) Correlations between two behaviors expressed at the adult stage, given that they are strongly correlated on the juvenile stage (r_JUV_ = 0.7), and according to the rank-order correlation of each trait across ontogeny (r_JUV-AD_) which is here varied from 0 to 0.9 and assumed to be the same for both traits. Plotted are the modal (dot) and 95% credible interval (bars) based on 1000 genotypes randomly drawn from a multivariate normal distribution. (b) Correlation between two traits across ages when the two traits show different patterns of GxA. Red line with dots is the pattern expected when both traits show a “fanning” pattern GxA (cf Fig. 2a), such that the ranking is maintained across ages. Blue line shows the expected correlation when one trait shows a “fanning” pattern and the other one a “crossing” pattern where the ranking is reversed across ages (cf. Fig. 2b). Black line shows the pattern expected when both traits have “crossing” GxA. Values plotted are based 1000 genotypes randomly drawn from a bivariate normal distribution with unity variance in elevation and slope and, for “fanning” GxA, a correlation of +0.9 between elevation and slope, and, for “crossing” GxA, a correlation of –0.9 (see the R script, additional file 4).

Theoretically, whenever the rank-order of individuals for a particular behavior changes over ages, the correlation of this behavior to another behavior (the behavioral syndrome) is expected to change as the organism ages [21,106]. We here illustrate this classic result quantitatively based on a simulation (additional file 4) assuming there are two behaviors expressed by individuals in two age classes (juvenile and adults). The focal behaviors are strongly positively correlated in the juvenile stage (r_JUV_=0.7). We calculate the expected correlation and its 95% credible interval between these behaviors in adults for 1000 genotypes randomly drawn from a multivariate normal distribution. We then assume that the correlation of each behavior across ages (r_JUV-AD_) varies from 0 (behavior in juveniles and in adults are completely uncorrelated) to 0.9 (behavior as a juvenile and an adult are highly correlated). Clearly, the expected correlation between the two behaviors in the adult stage r_AD_ is always lower than in the juvenile stage (because E(r_AD_) = (r_JUV-AD_)^2^ r_JUV_), as illustrated by the expected value (dots in Fig. 4). Furthermore, fairly strong cross-ontogeny correlations(exceeding 0.3)are required for the expected correlation between the behaviors in adults to become statistically distinguishable from zero based on the assumed parameters (Fig. 4). Thus, if GxA occurs, a behavioral syndrome is stable over ages only when the GxA mainly concerns changes in variance across ages (i.e. reaction norms “fan”; Fig. 2a,b) and each of the behaviors which form a syndrome are correlated highly across ages (Fig. 4). In particular, it is noteworthy in this example that even when behaviors show a “reasonably high” positive correlation of 0.3 – 0.5 across ages, which is clearly commonly observed at least on the phenotypic level (Fig. 3), the correlation in two behaviors falls from 0.7 in juveniles to a low of approximately 0.1 – 0.2 in adults. Thus, the (implicit) assumption that a behavioral syndrome stays stable over age classes essentially assumes that the behaviors forming the syndrome show little to no variation in plasticity over age classes.

When considering more than two ages, it becomes particularly clear how different patterns of changes in the correlation between behaviors during aging can be due to different types of GxA[106]. To illustrate this we considered three different scenarios of GxA on the correlation between two behaviors from age 1 to 10. In the first scenario, reaction norms “fan out” such that the rank orders of both behaviors are maintained across ages (cf. Fig 2a). Under these conditions, the correlation between the behaviors is high and positive across all ages (red line in Fig. 4b). In the second scenario, the rank order of behaviors is maintained for the first trait but is for the second trait reversed in old ages (crossing reaction norms, cf. Fig 2d). The correlation between the behaviors then becomes negative (blue line in Fig. 4b) after the point where the reaction norms of the second behavior are crossing, i.e. where the variance reaches it minimum (cf. Fig. 2d). Finally, when the rank order of both traits is reversed across ages, the correlation between the behaviors initially decreases towards zero before increasing again in older ages (black line in Fig. 4b). Given the rarity of negative phenotypic rank-order correlations in behaviors, these 3 scenarios can seem far from biological reality. However, the key point of this exercise is to demonstrate that crossing of the IxA and/or GxA reaction norms within the range of ages considered in at least one of two correlated behaviors leads to changes in the correlation between these two behaviors across ages. Whenever such crossing of reaction norms occurs and depending on which ages are considered in a study, one would predict that correlations between behaviors either disappear (e.g. blue and black lines for ages 1-5 in Fig. 4b) or appear (black line for ages 6-10 in Fig.4b) or show changes in sign or non-linear patterns (ages 1-10 in Fig.4b).

## Correlation between multiple traits across ages: literature overview

Theoretical considerations [21,106] reviewed above clearly predict that age-specific correlations between multiple behaviors are expected to change as the organism ages, whenever IxA and/or GxA is present in one or more of the behaviors. In order to investigate whether such patterns are found in the literature, we selected among the studies reviewed in the previous section an overview of the literature on changes in age-specific phenotypic correlations between behaviors. Although the limited amount of studies does not allow quantitative statements, there indeed are studies finding a change in the magnitude and sometimes even the sign of the correlation between two behaviors across ages (additional file 5). At present, we do not know the extent to which the observed correlational changes are caused by underlying GxA and to what extent they are due to non-heritable age-related factors. Nevertheless, changes in correlations between behaviors over age classes clearly occur in nature, and hence beg an explanation. Explicit study of the extent to which GxA is underlying such changes is likely valuable for gaining further insight into this interesting phenomenon.

## Future perspective

The take-home message of this paper is that when there is plasticity in behavior over ages, there may be variation in age-related plasticity between individuals (IxA) and/or genotypes (GxA). Whenever there is variation in plasticity, there is the clear expectation that repeatability and/or heritability of a focal behavior varies over ages. In addition, it is likely that the ranking of individual-specific and/or breeding values for the focal behavior change as a function of age, thereby leading to low cross-age correlations in behavior, and changes in the correlation between multiple behaviors. Thus, calculation of the repeatability or heritability of a behavior or the correlation between multiple behaviors while pooling all age groups may give a misleading impression of the consistency in behavior or a behavioral syndrome. Our literature overview, based on phenotypic measures, indeed illustrates that the consistency of behavioral differences between individuals across ages may be low, despite a behavior being repeatable when measured at different ages. We believe that changes in repeatability and heritability over ages as well as appreciation of cross-age correlations are of importance if we are to properly understand the sources of behavioral variation from a lifetime perspective.

We have outlined conceptual models, which illustrate why the presence of IxA and GxA is expected to lead to age-related changes in repeatability, heritability and low cross-age correlations in behavior. In addition, we briefly underlined the quantitative genetic approaches which provide accessible means to model age-related plastic changes in behavior on the individual and/or genetic levels. In general, there is a clear scope for increased uptake of quantitative genetic approaches in the study of behavior [107]. We believe the approaches outlined in this paper are applicable to various systems in which behavior is studied, and hope future studies will consider approaching their research from this perspective. It is the explicit empirical investigation of IxA and GxA in behavior which will inform us whether the theoretical concepts and patterns predicted by theory on plasticity indeed are present in behaviors.

## Declarations

The authors declare no competing interests. Publication costs for this article were funded by the German Research Foundation (FOR 1232) and the Open Access Publication Fund of Bielefeld and Muenster University.

## Authors’ contributions

JEB conceived the study and wrote the first draft. BC performed the literature search and compiled the literature estimates. JEB and BC jointly wrote the paper, and its final version is approved by both authors.

## Supplementary Material

Additional file 1 Glossary of terms used in this paper Click here for file

Additional file 2 Schematic illustration of how changes in the age-specific expression of behavior by an individual are modelled as deviations from the age-specific mean Click here for file

Additional file 3 Overview of literature estimates of the correlation of behavioral traits across age classes Click here for file

Additional file 4 R script for producing the simulations in this paper Click here for file

Additional file 5 Overview of the literature considering changes in the correlation between two or more behavioral traits across ages Click here for file
