# Supplementary material for: The importance of genotype-by-age interactions for the development of repeatable behavior and correlated behaviors over lifetime
Source: Front Zool. 2015 Aug 24;12(Suppl 1):S2. doi: 10.1186/1742-9994-12-S1-S2 (PMC4722339; doi:10.1186/1742-9994-12-S1-S2)
Supplement: Additional file 1 — Glossary of terms used in this paper [file 1742-9994-12-S1-S2-S1.pdf]

Additional file 1: Glossary of terms used in this paper

**Terms**	**Brief explanation**
additive genetic variance, V_A	The variance in breeding values present in the base population. Typically estimated by the covariance in trait values measured in relatives of a known degree. Depends on allele frequencies and is hence a population specific property.
animal model	Linear mixed model which uses pedigree-derived relatedness between all individuals to estimate the additive genetic (co)variance(s) for trait(s).
base population	Those individuals in the population which are not a descendant of any individual in the population. Base population individuals are assumed to be unrelated. Quantitative genetic parameters are typically calculated for the base population because the genetic (co)variances of focal trait(s) in these individuals are prior to selection on this trait in the population.
between-individual variance	The variance in individual-specific trait values. Typically estimated as the variance between subjects in a mixed model where the individual's identity is included as the random subject, in which case the between-individual variance is conditional upon the fixed effects. Can be loosely interpreted as the variance in the individual-specific mean trait values for individuals with two or more repeated measures.

breeding value	Summed effect of all loci in the genome of an individual on its expression of a trait. The breeding value denotes the expected trait expression of an individual in the absence of other effects on this trait. Typically expressed as deviation from the population mean. Can be age-specific.
character-state approach	An approach to describe plasticity, in which the expected trait value of an individual and breeding value is specific to each age or environment such that changes across *n* ages or environments are fully described by an $n \times n$ covariance matrix on the individual and genetic level respectively.
genetic dominance variance	Variance in summed dominance deviations across all loci in each genome/individual in the base population.
G×A	Genotype – Age interaction. Term to indicate that breeding values vary as a function of age. Presence of G×A shows that the rate of change in a trait over ages is a heritable property, and hence is evidence for additive genetic *variance in plasticity*.
heritability, h^2	The proportion of phenotypic variance explained by additive genetic variance. Typically interpreted as the degree offspring are expected to resemble their parents. Can be age-specific, in which case it refers to the resemblance in trait value between an offspring of age *A* relative to its parents at age *A*.

I×A	Individual –Age interaction. Term to indicate that individual-specific trait values vary as a function of age. Presence of I×A shows that individuals differ in their rate of change in a trait over ages, and hence between-individual *variance in plasticity*.
mixed model	A statistical model including both fixed (population-wide) effects and random (subject specific) effects. Typically solved using either REML or Bayesian approaches.
permanent environment effect	The non-heritable effect associated to an individual which is conserved across the repeated measures made on that individual. Examples include maternal effects and differences in local environmental conditions experienced by individuals.
phenotype	The measured trait value of an individual
phenotypic variance	Variance in the measures taken. Can be age-specific. May denote variance conditional on fixed effects. For mixed models often calculated as the sum of all variances from which the random effects are assumed to be drawn.
repeatability	Proportion of phenotypic variance explained by between-individual variance. Also known as Intra-Class Correlation (ICC).
residual variance, V_R	The variance in the difference between the observed trait value and the one predicted on the basis of the

	fixed and possibly random effects included in a statistical model. Includes variance caused by factors which were not included in the model as well as measurement error.
random regression	Statistical model allowing both between-subject variance in intercept (different elevations), and in subject-specific slopes over a continuous covariate (e.g. age). Also known as random slopes model.
reaction norm	An approach to describe plasticity, in which a function describes the expected trait value of a population or individual or breeding value as a continuous function of age or environment.
RRAM	Random Regression Animal Model. An animal model which includes random slopes thereby allowing breeding values to vary over ages and hence one approach to model G×A.
trait	Aspect of an individual that can be quantified directly, i.e. has a phenotype. Can be a behavior.
