# Supplementary material for: The importance of genotype-by-age interactions for the development of repeatable behavior and correlated behaviors over lifetime
Source: Front Zool. 2015 Aug 24;12(Suppl 1):S2. doi: 10.1186/1742-9994-12-S1-S2 (PMC4722339; doi:10.1186/1742-9994-12-S1-S2)
Supplement: Additional file 2 — Schematic illustration of how changes in the age-specific expression of behavior by an individual are modelled as deviations from the age-specific mean [file 1742-9994-12-S1-S2-S2.pdf]

Additional file 2: Schematic illustration of how changes in the age-specific expression of behavior by an individual are modelled as deviations from the age-specific mean

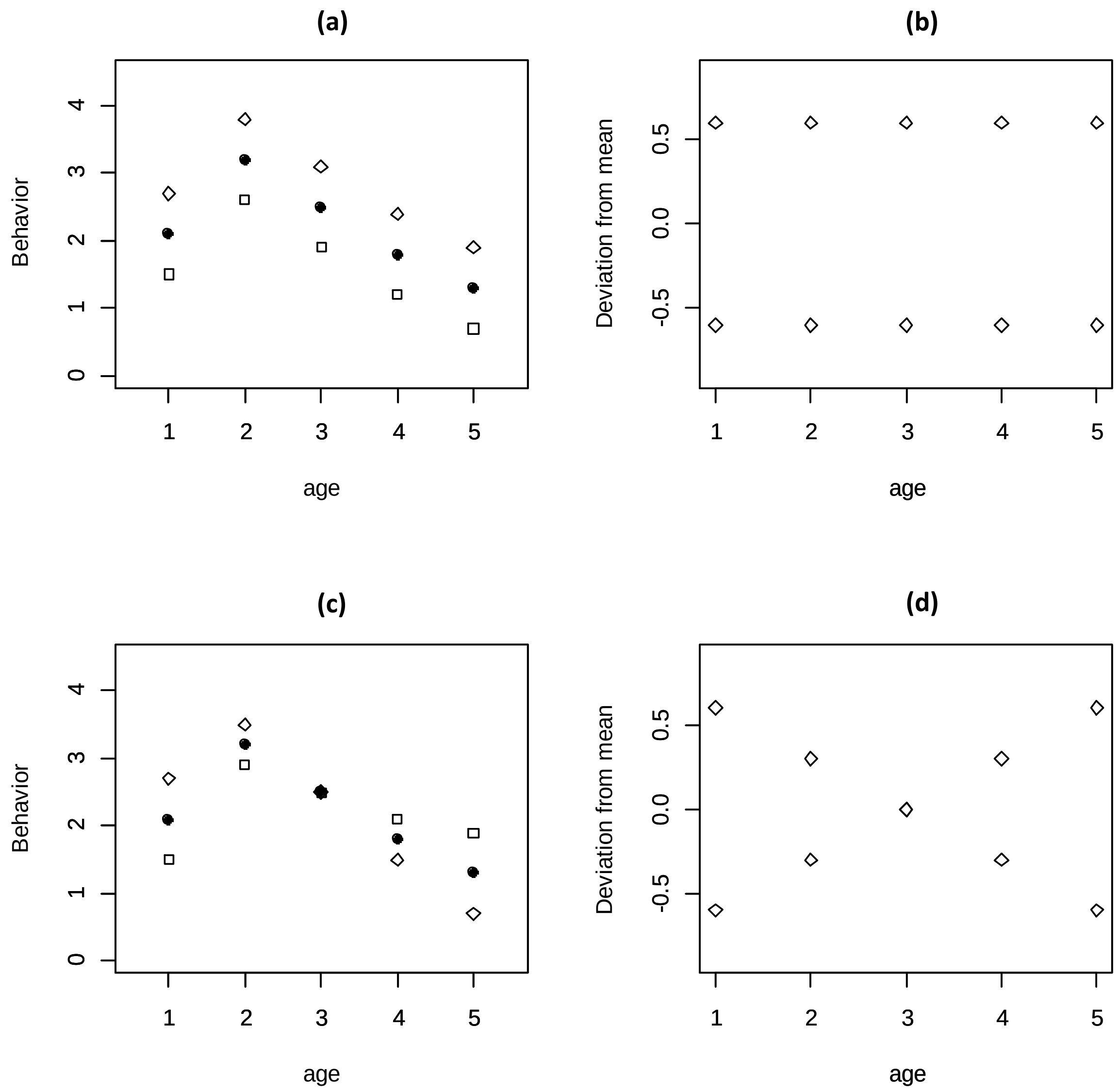

Panels (a,c) show the mean behavior over age (filled dots) and the behavior expressed by two individuals (diamond and square). Panels (b,d) show the individual behaviors as deviations from the age-specific mean. Character-state and random regression model concern plasticity after “factoring out” plasticity in the mean behavior, modelled as deviations from the fixed effect mean. Hence, panel (b) shows absence of IxA (individuals show the same plasticity), whereas panel (d) indicate presence of IxA (individuals differ in their plasticity).
