# Supplementary material for: The importance of genotype-by-age interactions for the development of repeatable behavior and correlated behaviors over lifetime
Source: Front Zool. 2015 Aug 24;12(Suppl 1):S2. doi: 10.1186/1742-9994-12-S1-S2 (PMC4722339; doi:10.1186/1742-9994-12-S1-S2)
Supplement: Additional file 3 — Overview of literature estimates of the correlation of behavioral traits across age classes [file 1742-9994-12-S1-S2-S3.pdf]

Additional file 3: Overview of literature estimates of the correlation of behavioral traits across age classes

Ref	Species	Origin	Ages	Traits	Cor 1-2	Cor 2-3	Cor 3-4	Cor 4-5	Cor 5-6
6	Pig	Breeding	5-7 and 10-12 weeks	human approach test	0.16				
				NO test	0.26				
				open door test	0.24				
40	Yellow-bellied marmots	Wild	juveniles, 1year old, >=2 years	docility	0.86	0.78			
				boldness	0.38	0.45			
41	Zebra finch	Lab	2 months, 263 days later	exploration	0.76				
			2 months, 209 days later	struggling rate	0.15				
42	Crayfish	Lab	~17 days, 125 days	boldness during the day	0.75				
43	Dumpling squid	Lab	3,6,9,12,16 weeks	shy/bold behavior in threat	0.26	0.42	0.15	0.54	
				shy/bold behavior in feeding	0.32	0.42	0.04	0.01	
44	Siberian dwarf hamster	Lab	juvenile (18–29 days), adult1 (45–65 days), and adult2(>72 days)	open field (OF) activiy	0.42	0.55			
				total time in open field	0.34	0.39			
				tunnel activity	0.57	0.74			
				tunnel boldness	-0.16	0.38			
				tunnel reactivity	-0.27	0.37			
				nest box orientation	0.18	0.11			
				open field escape	0.42	0.27			
				OF manipulate	0.27	0.16			
45	Great tit	Wild	first summer-first breeding season	dispersal distance females	0.87	0.72	0.91		
				dispersal distance males	0.65	0.72	0.85		
46	Pig	Breeding	46,80,113 days	attack latency	0.26	0.46			
47	Guinea pig	Lab	22days,10 days after maturation, 6 month	long field latency	0.26				
				distance OF	0.47				
				latency to touch novel object (NO)	0.00				
48	Guinea pig	Lab	22 days,shorty after maturation	fearlessness	0.36				
				boldness	0.23				
				exploration	0.23				
49	Rabbit (male)	Field enclosure	5-16 weeks , 16-22 weeks	aggressiveness	0.32				
				resident intruder test 1	0.34				
				resident intruder test 2	0.28				

Ref	Species	Origin	Ages	Traits	Cor 1-2	Cor 2-3	Cor 3-4	Cor 4-5	Cor 5-6
50	Pig	Breeding	8,24 weeks	resident intruder test 3	0.07				
				person approach test 1	0.33				
				person approach test 2	0.29				
				person approach test 3	0.28				
				person approach test 4	0.28				
				person approach test 5	-0.19				
				person approach test 6	0.02				
				NO test 1	0.53				
				NO test 2	0.44				
				NO test 3	0.28				
				NO test 4	0.30				
				NO test 5	0.05				
				NO test 6	0.10				
51	Pig	Breeding	10,24 weeks	aggression while feeding	0.61				
				nb escapeduring backtest (BT)	0.17				
				duration escape BT	0.21				
				nb vocalization BT	0.23				
				latency to leave pen NO	-0.06				
				locomotion corridor NO	0.21				
				latency human contact NO	0.01				
				cortisol response NO	-0.07				
52	Pig	Breeding	49,77 days	attack latency	0.57				
53	Stickleback	Wild Navarro river	juvenile,subadults,adults (98-322 days)	activity	0.17	-0.05			
				aggression	0.33	0.32			
				boldness	0.08	0.12			
		Wild Putah Creek	juvenile,subadults,adults (98-322 days)	activity	0.43	-0.38			
				aggression	0.70	-0.02			
				boldness	-0.02	0.73			
				flightiness (PCA)	0.49				
				sensitiveness (PCA)	0.02				
				NO test 1	0.22				
				NO test 2	0.24				

Ref	Species	Origin	Ages	Traits	Cor 1-2	Cor 2-3	Cor 3-4	Cor 4-5	Cor 5-6
54	Horse	Breeding	9,22 months	NO test 3	0.37				
				NO test 4	0.04				
				NO test 5	0.41				
				NO test 6	0.30				
				NO test 7	0.44				
				NO test 8	0.68				
				NO test 9	0.24				
				handling test 1	0.26				
				handling test 2	0.14				
				handling test 3	0.30				
				handling test 4	0.35				
				handling test 5	0.15				
				handling test 6	0.31				
				handling test 7	0.27				
				handling test 8	0.03				
				handling test 9	0.28				
55	Welsh pony	Breeding	8 months,1.5 years, 2.5 years	reactivity to humans (RH) test 1	0.43	0.45			
				RH test 2	0.42	0.60			
				RH test 3	NS	0.41			
				RH test 4	NS	0.38			
				RH test 5	NS	0.39			
				RH test 7	NS	0.57			
				RH test 8	0.40	0.58			
				RH test 9	0.45	0.50			
				RH test 10	NS	0.75			
				RH test 11	0.51	0.67			
				RH test 12	0.40	0.63			
				RH test 13	0.42	0.62			
				RH test 14	0.47	0.65			
				RH test 15	NS	0.48			
				RH test 16	0.41	NS			
				RH test 1	0.42	0.41			

Ref	Species	Origin	Ages	Traits	Cor 1-2	Cor 2-3	Cor 3-4	Cor 4-5	Cor 5-6
	Anglo Arab horse	Breeding	8 months,1.5 years, 2.5 years	RH test 6	NS	0.63			
				RH test 8	0.44	0.45			
				RH test 9	NS	0.39			
				RH test 10	NS	0.59			
				RH test 11	0.60	NS			
				RH test 12	0.68	NS			
				RH test 13	0.57	NS			
				RH test 14	0.58	NS			
				RH test 15	0.52	NS			
				RH test 17	0.45	NS			
56	Welsh pony	Breeding	8 months,1.5 years, 2.5 years	NO test 1	0.76	0.59			
				NO test 2	0.75	0.60			
				NO test 3	0.56	0.56			
				NO test 4	0.61	0.67			
				NO test 5	0.65	0.58			
				NO test 6	0.68	0.61			
				NO test 7	0.54	0.58			
				NE test 1	0.71	0.69			
				NE test 2	0.79	0.60			
				NE test 3	0.54	0.66			
				NE test 4	0.69	0.80			
				NE test 5	0.70	0.66			
				NE test 6	NS	0.38			
				NE test 7	NS	0.68			
				NE test 8	NS	0.51			
				NE test 9	NS	0.71			
				surprise test 1	0.35	0.61			
				surprise test 2	0.36	0.69			
				surprise test 3	NS	0.61			
				surprise test 4	NS	0.57			
				NO test 1	0.47	0.60			
				NO test 2	0.40	0.59			

Ref	Species	Origin	Ages	Traits	Cor 1-2	Cor 2-3	Cor 3-4	Cor 4-5	Cor 5-6
	Anglo Arab horse	Breeding	8 months,1.5 years, 2.5 years	NO test 3	0.46	0.70			
				NO test 4	0.43	0.45			
				NO test 5	0.51	0.67			
				NO test 6	0.53	0.61			
				NE test 1	0.61	0.66			
				NE test 2	0.55	0.46			
				NE test 3	0.53	0.65			
				NE test 4	0.55	NS			
				NE test 5	0.53	NS			
				NE test 6	0.48	NS			
				surprise test 1	0.62	0.79			
				surprise test 2	0.74	0.65			
				surprise test 3	0.58	0.68			
				surprise test 4	NS	0.50			
57	Welsh pony	Breeding	8 months,1.5 years, 2.5 years	isolation 1	0.41	NS			
				isolation 2	0.43	NS			
				isolation 3	NS	0.51			
				isolation 5	NS	0.47			
				isolation 6	NS	0.40			
				separation 1	0.41	0.53			
				separation 2	0.38	0.48			
				separation 3	NS	0.38			
				separation 4	0.48	NS			
				separation 5	NS	0.40			
				separation 8	0.40	0.39			
				passage 1	0.68	0.63			
				passage 2	0.37	0.47			
				passage 4	0.40	NS			
				isolation 1	0.45	NS			
				isolation 2	0.45	NS			
				isolation 4	0.46	NS			
				isolation 7	0.67	NS			

Ref	Species	Origin	Ages	Traits	Cor 1-2	Cor 2-3	Cor 3-4	Cor 4-5	Cor 5-6
	Anglo Arab horse	Breeding	8 months,1.5 years, 2.5 years	separation 1	0.46	0.55			
				separation 2	0.57	NS			
				separation 3	0.67	NS			
				separation 4	0.45	0.48			
				separation 5	0.64	NS			
				separation 6	NS	0.46			
				separation 7	NS	0.55			
				separation 9	NS	0.50			
				attraction 2	0.64	NS			
				attraction 3	0.47	NS			
				passage 1	0.49	0.56			
				passage 2	NS	0.46			
				passage 3	0.63	NS			
				passage 4	NS	0.43			
58	Rat	Lab	6,11,16,21,37,52 weeks	hole board test 1	0.65	0.67	0.71	0.75	0.54
				hole board test 2	0.55	0.54	0.69	0.63	0.66
				hole board test 3	0.67	0.81	0.80	0.80	0.84
				hole board test 4	0.41	0.80	0.87	0.65	0.70
				canopy test 1	0.55	0.60	0.48	0.64	0.22
				canopy test 2	0.63	0.65	0.64	0.76	0.74
				canopy test 3	0.34	0.55	0.68	0.70	0.58
				canopy test 4	0.52	0.96	0.99	0.99	0.99
59	Great tit fast line	Lab	juvenile, adults	NO test	0.31				
				novel environment	0.76				
	Great tit slow line	Lab	juvenile, adults	novel environment	0.26				
60	Cichlid fish	Lab	juveniles, adults	exploration	0.59				
				boldness	0.19				
				restraint aggression	0.02				
				overt aggression	0.43				
				agonistic behaviour 1	0.79	0.96			
				agonistic behaviour 2	0.57	0.88			

Ref	Species	Origin	Ages	Traits	Cor 1-2	Cor 2-3	Cor 3-4	Cor 4-5	Cor 5-6
61	Rhesus monkey	Lab	Early years (6-10), Middle years (11-15), Later years (16-20)	agonistic behaviour 3	-0.39	0.14			
				agonistic behaviour 4	0.89	0.93			
				agonistic behaviour 5	1.00	0.61			
				agonistic behaviour 6	0.68	0.07			
				social behaviour 1	0.54	0.89			
				social behaviour 2	0.14	0.39			
				social behaviour 3	0.81	0.61			
				social behaviour 4	0.93	0.11			
				social behaviour 5	0.86	0.68			
				social behaviour 6	0.53	0.96			
				social behaviour 7	0.82	0.86			
				locomotion	0.64	0.11			
				exploration	0.57	0.86			
				passive visual	0.86	1.00			
				object play	0.72	0.96			
				self-directed behaviour 1	0.54	0.11			
				self-directed behaviour 2	0.89	0.57			
				self-directed behaviour 3	0.89	0.75			
62	*Octopus bimaculoides*	Lab	3,6,9 weeks	active engagement (PCA)	-0.49	-0.53			
				arousal/readiness (PCA)	0.59	-0.72			
				aggression (PCA)	-0.54	-0.72			
				avoidance/disinterest (PCA)	-0.59	-0.07			
63	*Amazona amazonica*	Lab	Juveniles (12-32 months), adults (1 year later)	neuroticism	0.82				
				extraversion	0.88				
64	Marmoset	Lab	6,12,18 months	phee calling	0.67	0.42			
				alarm calling	0.03	-0.07			
				cage manipulations	0.49	0.54			
				locomotion	0.18	0.36			
65	Common voles	Lab	Over maturation (from 62 to 152 days)	latency	0.70				
				activity	0.21				
66	Lake frog	Lab	Across metamorphosis	activity/exploration	0.56				
67	Firebug	Lab	5th larval stage, adults	composite personality score	0.19				

Ref	Species	Origin	Ages	Traits	Cor 1-2	Cor 2-3	Cor 3-4	Cor 4-5	Cor 5-6
68	Raven	Lab	3 months, 6months	latency to approach to NO	0.65				
				duration of object manipulation	0.75				
				duration close to NO	0.51				
69	Heifer calves	Breeding	7 months, 22 months	locomotion during OF test	0.48				
				vocalizations during OF test	0.42				
70	Pig	Breeding	60, 95 and 130 days	attack latency	0.30	0.27			
71	Cichlid fish	Lab	4 -4.5 months, 12-13.5 months	locomotion OF	-0.07	0.13			
				strange fish test 1	0.19	-0.07			
				strange fish test 2	-0.33	0.78			
				strange fish test 3	0.01	0.54			
				strange fish test 4	0.17	0.56			
				mirror test 1	0.42	0.27			
				mirror test 2	0.13	0.64			
				mirror test 3	0.31	0.66			
72	Scrub jay	Wild	70 days, 1,2,3 years	stress-induced CORT	0.71	0.38	-0.04		
73	Heifer calves	Breeding	16 weeks, 18 months	nb of grid crossing	0.27				
74	Horse	Breeding	9,10,21,22 months	heart rate during NO test	0.51	0.50	0.43		
				sd of beat-to-beat interval NO test	0.19	0.24	0.46		
				the root mean square of successive beat-to-beat differences NO test	0.33	0.30	0.40		
				heart rate during handling test	0.61	0.38	0.53		
				sd of beat-to-beat interval handling test	0.22	0.46	0.70		
				the root mean square of successive beat-to-beat differences handling test	0.34	0.27	0.40		
75	Ground squirrel	Wild	76-77 days, 89-90 days	tail elevation towards snake	0.37				
				investigative approach towards snake	0.10				
				litter throwing towards snake	0.74				
				tail flagging activity towards snake	0.12				
				tail elevation towards cat	0.88				

Ref	Species	Origin	Ages	Traits	Cor 1-2	Cor 2-3	Cor 3-4	Cor 4-5	Cor 5-6
				investigative approach towards cat	0.10				
				litter throwing towards cat	0.95				
76	Midas cichlid	Lab	12-22 weeks, 29-37 weeks, 44-62 weeks, 62-82 weeks	mirror test 1	0.41	0.43	0.54	0.74	
				mirror test 2	0.33	0.43	0.57	0.69	
				mirror test 3	0.46	0.48	0.66	0.56	
77	Zebra finch	Lab	358 days , 985 days	approach to novel object score	0.45				

For each species, it is denoted whether the population studied was of lab, wild or animal breeding origin and at which ages which behaviors were quantified. The correlation in the behavior between successive age classes is denoted as “Cor age-age+1”. For instance, “Cor 1-2” is the correlation in behavior between the first and second age class considered. Correlations considered to be significantly non-zero by the autors are printed in boldface; correlations which were not reported because they were not significant are denoted as "NS". Note that certain studies reported many estimates of the "same" behavior (as indicated by numbering these behaviors in this table). The correlations for these behaviors were averaged for presentation in Fig. 3 in the main text.Reference to each study is made using a numeric index as stated under “Ref”, which is cited in the main text
