# Supplementary material for: The importance of genotype-by-age interactions for the development of repeatable behavior and correlated behaviors over lifetime
Source: Front Zool. 2015 Aug 24;12(Suppl 1):S2. doi: 10.1186/1742-9994-12-S1-S2 (PMC4722339; doi:10.1186/1742-9994-12-S1-S2)
Supplement: Additional file 4 — R script for producing the simulations in this paper [file 1742-9994-12-S1-S2-S4.pdf]

Additional file 4: R script for producing the simulations in this paper

```
### SECTION 1 ###
# R script to produce the simulated reaction norms for Figure 2 in
the main text. This script displays one realization.
# Written by JEB. Script is for illustrative purposes only
rm(list=ls())
#plotting of reaction norms variances and correlations
require(mvtnorm)
#
#function to plot the RNs
plotRN<-function(dat2plot,leftright) {
if (leftright==1){
      panel=c("(a)","(b)","(c)")
} else {
      panel=c("(d)","(e)","(f)")
}
yminmax=c(round(min(dat2plot))-1,round(max(dat2plot))+1)
plot(dat2plot[1,]~x,type="l",ylim=yminmax,
ylab=list("Behavior",cex=1.2), xlab=list("Age",
cex=1.2),main=panel[1])
for (i in 2:n) {
      par(new=T)
      plot(dat2plot[i,]~x,type="l",ylim=yminmax, ylab=NA, xlab=NA,
axes=F)
}
#plot vertical dotted lines at ages 2 and 4
lines(c(2,2),yminmax,lty="dashed")
lines(c(4,4),yminmax,lty="dashed")
#variance over ages
#par(new=F)
plot(apply(dat2plot,2,var)~x,type="b",ylab=list("Variance",cex=1.2),
xlab=list("Age",cex=1.2),main=panel[2])
#correlation between young and old
plot(dat2plot[,4]~dat2plot[,2],pch=19,ylab=list("Behavior at age
4",cex=1.2),xlab=list("Behavior at age
2",cex=1.2),main=panel[3],axes=T)
}
#¤¤¤¤¤¤¤¤¤¤¤¤¤¤¤¤¤¤¤¤¤¤¤¤¤¤¤¤¤¤¤¤¤¤¤¤¤¤¤¤¤¤¤¤¤¤¤¤¤¤¤¤¤¤¤¤¤¤¤¤¤¤¤¤¤¤
#generate some values; correlated elevation and slope
n=30
sigma <- matrix(c(4,1.3,1.3,1), ncol=2)
es <- rmvnorm(n=n, mean=c(0,0), sigma=sigma)
#plotting
layout(matrix(seq(6),3,2))
x<-seq(5)
dat2plot1<-matrix(es[,1],n,length(x))+
matrix(es[,2],n,length(x))*matrix((x-3),n,length(x),byrow=T)
plotRN(dat2plot1)
#generate some values; uncorrelated elevation and slope
sigma <- matrix(c(1,0,0,4), ncol=2)
es <- rmvnorm(n=n, mean=c(0,0), sigma=sigma)
#plotting
x<-seq(5)
dat2plot2<-matrix(es[,1],n,length(x))+
```

```
matrix(es[,2],n,length(x))*matrix((x-3),n,length(x),byrow=T)
plotRN(dat2plot2)

### SECTION 2 ###
# script for simulation of expected correlation in
# two correlated behaviors as a function of age (Fig 4)
# script written by Barbara Class
# the script if for illustration purposes only
#¤¤¤ construct a handy function for what we will do
library(mvtnorm)
getBiCop <- function(n, rho, X1) {
  C <- matrix(rho, nrow = 2, ncol = 2)
  diag(C) <- 1
  C <- chol(C)
  X2 <- rnorm(n, mean=mean(X1), sd=sd(X1))
  X <- cbind(X1,X2)
  # induce correlation (does not change X1)
  df <- X %*% C
  return(df[,2])
}

#¤¤¤¤¤¤¤¤¤¤¤¤¤¤¤¤¤ SIMULATION 1 ##############
#Simulation: rank order correlation of both traits vary from 0 to
0.9 across ontogeny
BS.onto<-array(0, dim= c(1000,4,10))
cross<-seq(from=0,to=0.9, by= 0.1)
rho=0.7
for (y in 1:10){
  for (i in 1:1000){
    #Generate data for trait 1 at age 1 and 2

    T1age1<-data.frame("Age"= "Age1", "Trait" =rnorm(1000),
"ID"=seq(1:1000))
    T1age1$rank<-rank(T1age1$Trait)
    T1age1<-T1age1[order(T1age1$Trait),]

    rankt1<- getBiCop(1000,cross[y],T1age1$rank)
    rankt1int<-rank(rankt1)

    T1age2<-data.frame("Age"= "Age2", "Trait"=rnorm(1000),
"ID"=NA,"rank"=NA)
    T1age2$rank=rank(T1age2$Trait)
    T1age2<-T1age2[(order(T1age2$rank)[rankt1int]),]

    BS1<-data.frame("T1A1"= T1age1$Trait ,"T1A2"= T1age2$Trait,
"ID"=T1age1$ID )

    #Generate data for T2 at age 1 and 2, T1 and T2 are correlated
at age 1 (rho=0.7)

    T2age1<-data.frame("Age"= "Age1", "Trait" = getBiCop(1000, rho,
T1age1$Trait), "ID"=NA)
    BS2<-data.frame("T1A1"= T1age1$Trait ,"T2A1"= T2age1$Trait,
"ID"=T1age1$ID )
```

```
    BS2$rankT2A1<-rank(BS2$T2A1)
    BS2<-BS2[order(BS2$T2A1),]

    rankt2<- getBiCop(1000,cross[y],BS2$rankT2A1)
    rankt2int<-rank(rankt2)

    T2age2<-data.frame("Age"= "Age2", "Trait"=rnorm(1000),
"ID"=NA,"rank"=NA)
    T2age2$rank=rank(T2age2$Trait)
    T2age2<-T2age2[(order(T2age2$rank)[rankt2int]),]

    BS2$T2A2<-T2age2$Trait

    BS2<-BS2[order(BS2$ID),]
    BS1<-BS1[order(BS1$ID),]

    BS<-data.frame("T1A1"= BS1$T1A1 ,"T1A2"= BS1$T1A2
,"T2A1"=BS2$T2A1,  "T2A2"=BS2$T2A2, "ID"=BS2$ID )


    BS.onto[i, 1,y]<-
cor.test(BS$T1A1,BS$T2A1,method="spearman")$estimate
    BS.onto[i, 2,y]<-
cor.test(BS$T1A2,BS$T2A2,method="spearman")$estimate
    BS.onto[i, 3,y]<-
cor.test(BS$T1A1,BS$T1A2,method="spearman")$estimate
    BS.onto[i, 4,y]<-
cor.test(BS$T2A1,BS$T2A2,method="spearman")$estimate
  }
}

require(MCMCglmm)

results.sim<-data.frame("Spearman corr"=NA,
"BS1"=NA,"BS1inf"=NA,"BS1sup"=NA, "BS2"=NA,"BS2inf"=NA,"BS2sup"=NA,
"BS3"=NA,"BS3inf"=NA,"BS3sup"=NA, "BS4"=NA,"BS4inf"=NA,"BS4sup"=NA)

for (i in 1:10){

  results.sim[i,1]<-cross[i]
  results.sim[i,2]<-
as.numeric(posterior.mode(as.mcmc((BS.onto[,1,i]))))
  results.sim[i,3]<-
as.numeric(HPDinterval(as.mcmc((BS.onto[,1,i])),0.95))[1]
  results.sim[i,4]<-
as.numeric(HPDinterval(as.mcmc((BS.onto[,1,i])),0.95))[2]
  results.sim[i,5]<-
as.numeric(posterior.mode(as.mcmc((BS.onto[,2,i]))))
  results.sim[i,6]<-
as.numeric(HPDinterval(as.mcmc((BS.onto[,2,i])),0.95))[1]
  results.sim[i,7]<-
as.numeric(HPDinterval(as.mcmc((BS.onto[,2,i])),0.95))[2]
  results.sim[i,8]<-
as.numeric(posterior.mode(as.mcmc((BS.onto[,3,i]))))
  results.sim[i,9]<-
as.numeric(HPDinterval(as.mcmc((BS.onto[,3,i])),0.95))[1]
```

```
  results.sim[i,10]<-
as.numeric(HPDinterval(as.mcmc((BS.onto[,3,i])),0.95))[2]
  results.sim[i,11]<-
as.numeric(posterior.mode(as.mcmc((BS.onto[,4,i]))))
  results.sim[i,12]<-
as.numeric(HPDinterval(as.mcmc((BS.onto[,4,i])),0.95))[1]
  results.sim[i,13]<-
as.numeric(HPDinterval(as.mcmc((BS.onto[,4,i])),0.95))[2]
}


#¤¤¤¤¤¤¤¤¤¤¤¤¤¤¤¤¤ SIMULATION 2 ¤¤¤¤¤¤¤¤¤¤¤¤¤¤¤¤¤
#Generate random values for 1000 individuals for each scenario

#First scenario: rank orders do not change for both traits, T1 and
T2 are correlated (rho=0.7) at age 1

#Trait 1:

T1age1<-data.frame("Age"= "Age1", "Trait" =rnorm(1000),
"ID"=seq(1:1000))

slope1<-getBiCop(1000, 0.9, T1age1$Trait)

T1age2<-data.frame("Age"= "Age2", "Trait" =T1age1$Trait+(slope1/9),
"ID"=seq(1:1000))
T1age3<-data.frame("Age"= "Age3", "Trait"
=T1age1$Trait+(slope1*(2/9)), "ID"=seq(1:1000))
T1age4<-data.frame("Age"= "Age4", "Trait"
=T1age1$Trait+(slope1*(3/9)), "ID"=seq(1:1000))
T1age5<-data.frame("Age"= "Age5", "Trait"
=T1age1$Trait+(slope1*(4/9)), "ID"=seq(1:1000))
T1age6<-data.frame("Age"= "Age6", "Trait"
=T1age1$Trait+(slope1*(5/9)), "ID"=seq(1:1000))
T1age7<-data.frame("Age"= "Age7", "Trait"
=T1age1$Trait+(slope1*(6/9)), "ID"=seq(1:1000))
T1age8<-data.frame("Age"= "Age8", "Trait"
=T1age1$Trait+(slope1*(7/9)), "ID"=seq(1:1000))
T1age9<-data.frame("Age"= "Age9", "Trait"
=T1age1$Trait+(slope1*(8/9)), "ID"=seq(1:1000))
T1age10<-data.frame("Age"= "Age10", "Trait" =T1age1$Trait+(slope1),
"ID"=seq(1:1000))

#Trait 2:

T2age1<-data.frame("Age"= "Age1", "Trait" = getBiCop(1000, 0.7,
T1age1$Trait ), "ID"=seq(1:1000))

slope2<-getBiCop(1000, 0.9, T2age1$Trait)

T2age2<-data.frame("Age"= "Age2", "Trait" =T2age1$Trait+(slope2/9),
"ID"=seq(1:1000))
T2age3<-data.frame("Age"= "Age3", "Trait"
=T2age1$Trait+(slope2*(2/9)), "ID"=seq(1:1000))
T2age4<-data.frame("Age"= "Age4", "Trait"
=T2age1$Trait+(slope2*(3/9)), "ID"=seq(1:1000))
```

```
T2age5<-data.frame("Age"= "Age5", "Trait"
=T2age1$Trait+(slope2*(4/9)), "ID"=seq(1:1000))
T2age6<-data.frame("Age"= "Age6", "Trait"
=T2age1$Trait+(slope2*(5/9)), "ID"=seq(1:1000))
T2age7<-data.frame("Age"= "Age7", "Trait"
=T2age1$Trait+(slope2*(6/9)), "ID"=seq(1:1000))
T2age8<-data.frame("Age"= "Age8", "Trait"
=T2age1$Trait+(slope2*(7/9)), "ID"=seq(1:1000))
T2age9<-data.frame("Age"= "Age9", "Trait"
=T2age1$Trait+(slope2*(8/9)), "ID"=seq(1:1000))
T2age10<-data.frame("Age"= "Age10", "Trait" =T2age1$Trait+(slope2),
"ID"=seq(1:1000))

#Calculate the correlations between Trait 1 and 2 at different ages
BSage1<-
cbind("age1"=cor.test(T1age1$Trait,T2age1$Trait,method="spearman")$e
stimate,

"age2"=cor.test(T1age2$Trait,T2age2$Trait,method="spearman")$estimat
e,

"age3"=cor.test(T1age3$Trait,T2age3$Trait,method="spearman")$estimat
e,

"age4"=cor.test(T1age4$Trait,T2age4$Trait,method="spearman")$estimat
e,

"age5"=cor.test(T1age5$Trait,T2age5$Trait,method="spearman")$estimat
e,

"age6"=cor.test(T1age6$Trait,T2age6$Trait,method="spearman")$estimat
e,

"age7"=cor.test(T1age7$Trait,T2age7$Trait,method="spearman")$estimat
e,

"age8"=cor.test(T1age8$Trait,T2age8$Trait,method="spearman")$estimat
e,

"age9"=cor.test(T1age9$Trait,T2age9$Trait,method="spearman")$estimat
e,

"age10"=cor.test(T1age10$Trait,T2age10$Trait,method="spearman")$esti
mate)

#Calculate variances for each trait across ontogeny

ontoT1S1<-
cbind(T1age1$Trait,T1age2$Trait,T1age3$Trait,T1age4$Trait,T1age5$Tra
it,

T1age6$Trait,T1age7$Trait,T1age8$Trait,T1age9$Trait,T1age10$Trait)

ontoT1S1<-as.data.frame(ontoT1S1)
varianceT1S1<-apply(ontoT1S1,2,var)
```

```
ontoT2S1<-
cbind(T2age1$Trait,T2age2$Trait,T2age3$Trait,T2age4$Trait,T2age5$Tra
it,

T2age6$Trait,T2age7$Trait,T2age8$Trait,T2age9$Trait,T2age10$Trait)

ontoT2S1<-as.data.frame(ontoT2S1)
varianceT2S1<-apply(ontoT2S1,2,var)


#Second scenario: rank orders crossed for Trait 2, not for Trait 1


T1age1<-data.frame("Age"= "Age1", "Trait" =rnorm(1000),
"ID"=seq(1:1000))

slope1<-getBiCop(1000, 0.9, T1age1$Trait)

T1age2<-data.frame("Age"= "Age2", "Trait" =T1age1$Trait+(slope1/9),
"ID"=seq(1:1000))
T1age3<-data.frame("Age"= "Age3", "Trait"
=T1age1$Trait+(slope1*(2/9)), "ID"=seq(1:1000))
T1age4<-data.frame("Age"= "Age4", "Trait"
=T1age1$Trait+(slope1*(3/9)), "ID"=seq(1:1000))
T1age5<-data.frame("Age"= "Age5", "Trait"
=T1age1$Trait+(slope1*(4/9)), "ID"=seq(1:1000))
T1age6<-data.frame("Age"= "Age6", "Trait"
=T1age1$Trait+(slope1*(5/9)), "ID"=seq(1:1000))
T1age7<-data.frame("Age"= "Age7", "Trait"
=T1age1$Trait+(slope1*(6/9)), "ID"=seq(1:1000))
T1age8<-data.frame("Age"= "Age8", "Trait"
=T1age1$Trait+(slope1*(7/9)), "ID"=seq(1:1000))
T1age9<-data.frame("Age"= "Age9", "Trait"
=T1age1$Trait+(slope1*(8/9)), "ID"=seq(1:1000))
T1age10<-data.frame("Age"= "Age10", "Trait" =T1age1$Trait+(slope1),
"ID"=seq(1:1000))


#Generate values for Trait2

T2age1<-data.frame("Age"= "Age1", "Trait" = getBiCop(1000, 0.7,
T1age1$Trait), "ID"=NA)
BS2<-data.frame("T1A1"= T1age1$Trait ,"T2A1"= T2age1$Trait,
"ID"=T1age1$ID )


BS2$rankT2A1<-rank(BS2$T2A1)
BS2<-BS2[order(BS2$T2A1),]

rankt2<- getBiCop(1000,-0.9,BS2$rankT2A1)
rankt2int<-rank(rankt2)

T2age10<-data.frame("Age"= "Age2", "Trait"=rnorm(1000),
"ID"=NA,"rank"=NA)
T2age10$rank=rank(T2age10$Trait)
T2age10<-T2age10[(order(T2age10$rank)[rankt2int]),]
```

```
BS2$T2A2<-T2age10$Trait
BS2<-BS2[order(BS2$ID),]


Trait2<-BS2[,c(2,5,3)]
Trait2$Slope<- Trait2[,2]-Trait2[,1]

T2age1<-data.frame("Age"= "Age1", "Trait" =Trait2[,1],
"ID"=Trait2[,3])
T2age2<-data.frame("Age"= "Age2", "Trait"
=Trait2[,1]+(Trait2$Slope/9), "ID"=BS2[,3])
T2age3<-data.frame("Age"= "Age3", "Trait"
=Trait2[,1]+(Trait2$Slope*(2/9)), "ID"=BS2[,3])
T2age4<-data.frame("Age"= "Age4", "Trait"
=Trait2[,1]+(Trait2$Slope*(3/9)), "ID"=BS2[,3])
T2age5<-data.frame("Age"= "Age5", "Trait"
=Trait2[,1]+(Trait2$Slope*(4/9)), "ID"=BS2[,3])
T2age6<-data.frame("Age"= "Age6", "Trait"
=Trait2[,1]+(Trait2$Slope*(5/9)), "ID"=BS2[,3])
T2age7<-data.frame("Age"= "Age7", "Trait"
=Trait2[,1]+(Trait2$Slope*(6/9)), "ID"=BS2[,3])
T2age8<-data.frame("Age"= "Age8", "Trait"
=Trait2[,1]+(Trait2$Slope*(7/9)), "ID"=BS2[,3])
T2age9<-data.frame("Age"= "Age9", "Trait"
=Trait2[,1]+(Trait2$Slope*(8/9)), "ID"=BS2[,3])
T2age10<-data.frame("Age"= "Age10", "Trait" =Trait2[,2],
"ID"=Trait2[,3])

#Calculate the correlations between Trait 1 and 2 at different ages

BSage2<-
cbind("age1"=cor.test(T1age1$Trait,T2age1$Trait,method="spearman")$e
stimate,

"age2"=cor.test(T1age2$Trait,T2age2$Trait,method="spearman")$estimat
e,

"age3"=cor.test(T1age3$Trait,T2age3$Trait,method="spearman")$estimat
e,

"age4"=cor.test(T1age4$Trait,T2age4$Trait,method="spearman")$estimat
e,

"age5"=cor.test(T1age5$Trait,T2age5$Trait,method="spearman")$estimat
e,

"age6"=cor.test(T1age6$Trait,T2age6$Trait,method="spearman")$estimat
e,

"age7"=cor.test(T1age7$Trait,T2age7$Trait,method="spearman")$estimat
e,

"age8"=cor.test(T1age8$Trait,T2age8$Trait,method="spearman")$estimat
e,
```

```
"age9"=cor.test(T1age9$Trait,T2age9$Trait,method="spearman")$estimat
e,

"age10"=cor.test(T1age10$Trait,T2age10$Trait,method="spearman")$esti
mate)

#Calculate variances for each trait across ontogeny

ontoT1S2<-
cbind(T1age1$Trait,T1age2$Trait,T1age3$Trait,T1age4$Trait,T1age5$Tra
it,

T1age6$Trait,T1age7$Trait,T1age8$Trait,T1age9$Trait,T1age10$Trait)

ontoT1S2<-as.data.frame(ontoT1S2)
varianceT1S2<-apply(ontoT1S2,2,var)

ontoT2S2<-
cbind(T2age1$Trait,T2age2$Trait,T2age3$Trait,T2age4$Trait,T2age5$Tra
it,

T2age6$Trait,T2age7$Trait,T2age8$Trait,T2age9$Trait,T2age10$Trait)

ontoT2S2<-as.data.frame(ontoT2S2)
varianceT2S2<-apply(ontoT2S2,2,var)


#Scenario 3: Rank orders changed for both traits

#Trait1

T1age1<-data.frame("Age"= "Age1", "Trait" =rnorm(1000),
"ID"=seq(1:1000))
T1age1$rank<-rank(T1age1$Trait)
T1age1<-T1age1[order(T1age1$Trait),]

rankt1<- getBiCop(1000,-0.9,T1age1$rank)
rankt1int<-rank(rankt1)

T1age10<-data.frame("Age"= "Age10", "Trait"=rnorm(1000),
"ID"=NA,"rank"=NA)
T1age10$rank=rank(T1age10$Trait)
T1age10<-T1age10[(order(T1age10$rank)[rankt1int]),]

BS1<-data.frame("T1A1"= T1age1$Trait ,"T1A10"= T1age10$Trait,
"ID"=T1age1$ID )


#Trait 2

T2age1<-data.frame("Age"= "Age1", "Trait" = getBiCop(1000, 0.7,
T1age1$Trait), "ID"=NA)
BS2<-data.frame("T1A1"= T1age1$Trait ,"T2A1"= T2age1$Trait,
"ID"=T1age1$ID )
```

```
BS2$rankT2A1<-rank(BS2$T2A1)
BS2<-BS2[order(BS2$T2A1),]

rankt2<- getBiCop(1000,-0.9,BS2$rankT2A1)
rankt2int<-rank(rankt2)

T2age10<-data.frame("Age"= "Age10", "Trait"=rnorm(1000),
"ID"=NA,"rank"=NA)
T2age10$rank=rank(T2age10$Trait)
T2age10<-T2age10[(order(T2age10$rank)[rankt2int]),]

BS2$T2A10<-T2age10$Trait

BS2<-BS2[order(BS2$ID),]
BS1<-BS1[order(BS1$ID),]

BS<-data.frame("T1A1"= BS1$T1A1 ,"T1A10"= BS1$T1A10
,"T2A1"=BS2$T2A1,  "T2A10"=BS2$T2A10, "ID"=BS2$ID )

#Calculate values for ages between 1 and 10 for Trait 1

Trait1<-BS[,c(1:2,5)]
Trait1$Slope<- Trait1[,2]-Trait1[,1]

T1age1<-data.frame("Age"= "Age1", "Trait" =Trait1[,1],
"ID"=Trait1[,3])
T1age2<-data.frame("Age"= "Age2", "Trait"
=Trait1[,1]+(Trait1$Slope/9), "ID"=Trait1[,3])
T1age3<-data.frame("Age"= "Age3", "Trait"
=Trait1[,1]+(Trait1$Slope*(2/9)), "ID"=Trait1[,3])
T1age4<-data.frame("Age"= "Age4", "Trait"
=Trait1[,1]+(Trait1$Slope*(3/9)), "ID"=Trait1[,3])
T1age5<-data.frame("Age"= "Age5", "Trait"
=Trait1[,1]+(Trait1$Slope*(4/9)), "ID"=Trait1[,3])
T1age6<-data.frame("Age"= "Age6", "Trait"
=Trait1[,1]+(Trait1$Slope*(5/9)), "ID"=Trait1[,3])
T1age7<-data.frame("Age"= "Age7", "Trait"
=Trait1[,1]+(Trait1$Slope*(6/9)), "ID"=Trait1[,3])
T1age8<-data.frame("Age"= "Age8", "Trait"
=Trait1[,1]+(Trait1$Slope*(7/9)), "ID"=Trait1[,3])
T1age9<-data.frame("Age"= "Age9", "Trait"
=Trait1[,1]+(Trait1$Slope*(8/9)), "ID"=Trait1[,3])
T1age10<-data.frame("Age"= "Age10", "Trait" =Trait1[,2],
"ID"=Trait1[,3])


#The same for Trait 2

Trait2<-BS[,c(3:5)]
Trait2$Slope<- Trait2[,2]-Trait2[,1]

T2age1<-data.frame("Age"= "Age1", "Trait" =Trait2[,1],
"ID"=Trait2[,3])
T2age2<-data.frame("Age"= "Age2", "Trait"
=Trait2[,1]+(Trait2$Slope/9), "ID"=Trait2[,3])
```

```
T2age3<-data.frame("Age"= "Age3", "Trait"
=Trait2[,1]+(Trait2$Slope*(2/9)), "ID"=Trait2[,3])
T2age4<-data.frame("Age"= "Age4", "Trait"
=Trait2[,1]+(Trait2$Slope*(3/9)), "ID"=Trait2[,3])
T2age5<-data.frame("Age"= "Age5", "Trait"
=Trait2[,1]+(Trait2$Slope*(4/9)), "ID"=Trait2[,3])
T2age6<-data.frame("Age"= "Age6", "Trait"
=Trait2[,1]+(Trait2$Slope*(5/9)), "ID"=Trait2[,3])
T2age7<-data.frame("Age"= "Age7", "Trait"
=Trait2[,1]+(Trait2$Slope*(6/9)), "ID"=Trait2[,3])
T2age8<-data.frame("Age"= "Age8", "Trait"
=Trait2[,1]+(Trait2$Slope*(7/9)), "ID"=Trait2[,3])
T2age9<-data.frame("Age"= "Age9", "Trait"
=Trait2[,1]+(Trait2$Slope*(8/9)), "ID"=Trait2[,3])
T2age10<-data.frame("Age"= "Age10", "Trait" =Trait2[,2],
"ID"=Trait2[,3])

#Calculate the correlations between Trait 1 and 2 at different ages

BSage3<-
cbind("age1"=cor.test(T1age1$Trait,T2age1$Trait,method="spearman")$e
stimate,

"age2"=cor.test(T1age2$Trait,T2age2$Trait,method="spearman")$estimat
e,

"age3"=cor.test(T1age3$Trait,T2age3$Trait,method="spearman")$estimat
e,

"age4"=cor.test(T1age4$Trait,T2age4$Trait,method="spearman")$estimat
e,

"age5"=cor.test(T1age5$Trait,T2age5$Trait,method="spearman")$estimat
e,

"age6"=cor.test(T1age6$Trait,T2age6$Trait,method="spearman")$estimat
e,

"age7"=cor.test(T1age7$Trait,T2age7$Trait,method="spearman")$estimat
e,

"age8"=cor.test(T1age8$Trait,T2age8$Trait,method="spearman")$estimat
e,

"age9"=cor.test(T1age9$Trait,T2age9$Trait,method="spearman")$estimat
e,

"age10"=cor.test(T1age10$Trait,T2age10$Trait,method="spearman")$esti
mate)

#Calculate variances for each trait across ontogeny

ontoT1S3<-
cbind(T1age1$Trait,T1age2$Trait,T1age3$Trait,T1age4$Trait,T1age5$Tra
it,
```

```
T1age6$Trait,T1age7$Trait,T1age8$Trait,T1age9$Trait,T1age10$Trait)

ontoT1S3<-as.data.frame(ontoT1S3)
varianceT1S3<-apply(ontoT1S3,2,var)

ontoT2S3<-
cbind(T2age1$Trait,T2age2$Trait,T2age3$Trait,T2age4$Trait,T2age5$Tra
it,

T2age6$Trait,T2age7$Trait,T2age8$Trait,T2age9$Trait,T2age10$Trait)
ontoT2S3<-as.data.frame(ontoT2S3)

varianceT2S3<-apply(ontoT2S3,2,var)


#Plot the results
#¤¤¤ simulation 1 in panel a, 2 in panel b
layout(matrix(c(1,2),1,2,byrow=TRUE))
#panel a
# we plot the results of the second behavioral syndrome
y<-results.sim$BS2
x<-seq(from=0, to =0.9, by=0.1)
sdinf<-results.sim$BS2inf
sdsup<-results.sim$BS2sup
plot(x,y,main="(a)", xlab= list("Correlation juvenile - adult",
cex=1.3),ylab=list("Correlation between traits",cex=1.3),ylim=c(-
0.1,0.7), pch=16,cex=2)
arrows(x, sdinf, x, sdsup, code=3, angle=90, length=0.1)
lines(c(-0.2,0.95),c(0,0),lty=2)
#¤¤¤ panel b
x<-seq(1:10)
plot(as.vector(BSage1)~x,type="b",lwd=3,col="red",lty=1,main="(b)"
,xlab=list(" Age ",cex=1.3),ylab=list(" Correlation between traits
",cex=1.3),ylim=c(-0.6,0.8),xlim=c(1,10), cex.lab=1,cex.axis=1)
par(new=T)
plot(as.vector(BSage2)~x,type="b",col="blue",lwd=3,lty=1,xlab=NA,yla
b=NA,ylim=c(-0.6,0.8),xlim=c(1,10), cex.lab=1,cex.axis=1)
par(new=T)
plot(as.vector(BSage3)~x,type="b",col="black",lwd=3,lty=1,xlab=NA,yl
ab=NA,ylim=c(-0.6,0.8),xlim=c(1,10), cex.lab=1,cex.axis=1)
```
