# Supplementary material for: The importance of genotype-by-age interactions for the development of repeatable behavior and correlated behaviors over lifetime
Source: Front Zool. 2015 Aug 24;12(Suppl 1):S2. doi: 10.1186/1742-9994-12-S1-S2 (PMC4722339; doi:10.1186/1742-9994-12-S1-S2)
Supplement: Additional file 5 — Overview of the literature considering changes in the correlation between two or more behavioral traits across ages [file 1742-9994-12-S1-S2-S5.pdf]

Additional file 5: Overview of the literature considering changes in the correlation between two or more behavioral traits across ages

Species	Time-span	Behavioral syndrome	Cor age 1	Cor age 2	Cor age 3	Cor age 4	Change	Ref
Pig	5-7 weeks, 10-12 weeks	backtest-human approach	0.79	NS			disappears	6
Green lizard	juvenile, adult	time until body emergence-number of grid changes	0.09	0.49			appears at maturation	83
Green lizard	juvenile, adult	number of grid changes-time until head emergence	-0.04	0.34			appears at maturation	83
Siberian dwarf hamster	Juvenile (18–29 days), adult1 (45–65 days), and adult2(>72 days)	tunnel boldness-tunnel activity	0.64	0	-0.41		changes sign	44
Siberian dwarf hamster	Juvenile (18–29 days), adult1 (45–65 days), and adult2(>72 days)	open field orientation-open field activity	0	-0.51	-0.4		appears in adults	44
Siberian dwarf hamster	Juvenile (18–29 days), adult1 (45–65 days), and adult2(>72 days)	tunnel boldness-open field activity	0.12	0	-0.35		appears after maturation	44
Great tit	1st summer-autumn-winter-1st Breeding	exploration-dispersal	0.42	0.7	0.27	0.37	disappears-reappears	45
Guinea pig	22 days (juvenile),10 days after maturation, 6 months(adult)	latency long field-distance open field	0.34	0.02	0.26		disappears	47
Guinea pig	22 days (juvenile),10 days after maturation, 6 months(adult)	nb trips long field-distance open field	0.33	0.11	0.13		disappears	47
Guinea pig	22 days, 80 days	boldness-resting metabolic rate	-0.49	0			disappears	48
Guinea pig	22 days, 80 days	exploration-cortisol	0.45	0			disappears	48

Guinea pig	22 days, 80 days	boldness-exploration	0	0.45			appears at maturation	48
Pig	3 weeks,8 weeks, 24 weeks	duration immobility (3w)-human approach test (standing duration 8 and 24w)	-0.25	-0.04			disappears	50
Pig	3 weeks,8 weeks, 24 weeks	duration immobility(3w)-human approach test (walking duration 8 and 24w)	0.29	-0.05			disappears	50
Stickleback	Juvenile, subadult, adult (137 days, 210 days, 322 days)	activity-aggression	0.65	0.26	0.38		disappears-reappears	53
Stickleback	Juvenile, subadult, adult (137 days, 210 days, 322 days)	boldness-aggression	0.43	0.12	0.18		disappears	53
Stickleback	Juvenile, subadult, adult (137 days, 210 days, 322 days)	boldness-activity	0.48	-0.02	0.37		disappears-reappears	53
Cichlid fish	Juvenile, adult	exploration—sand carrying	0.43	0.14			disappears	60
Marmoset	6 months, 12 months, 18 months	alarm calling-cortisol reactivity	0.32	0.11	-0.07		disappears	64
Marmoset	6 months, 12 months, 18 months	cage manipulation- post-stressor cortisol regulation	-0.18	0.15	0.4		appears at maturation	64
Marmoset	6 months, 12 months, 18 months	locomotion- post-stressor cortisol regulation	0.18	0.28	0.49		appears at maturation	64
Lake frog	larva, adult	total activity (T1)-latency to 1st movement (T1)	-0.85	NS			disappears	66
Lake frog	larva, adult	total activity (T2)-latency to resume activity (T2)	-0.36	0.61			changes sign	66
Lake frog	larva, adult	total activity (T2)-latency to resume activity (T1)	-0.46	0.48			changes sign	66

Lake frog	larva, adult	total activity (T1)-latency to resume activity (T1)	NS	0.39			appears at maturation	66
Lake frog	larva, adult	latency to 1st movement (T2)-latency to resume activity (T2)	NS	-0.41			appears at maturation	66
Lake frog	larva, adult	total activity (T1)-latency to resume activity (T2)	NS	0.55			appears at maturation	66
Lake frog	larva, adult	flight initiation distance (T1)-total activity (T2)	NS	0.49			appears at maturation	66
Lake frog	larva, adult	latency to 1st movement (T1)-latency to resume activity (T2)	NS	-0.44			appears at maturation	66

For each species, the ages at which the correlation was quantified is given, as well as the behaviors for which a correlation was calculated. Some studies calculated correlations for multiple traits and these are presented here separately. For each age class, a correlation is given: thus, a study which considered two age classes has two correlations reported such that “Cor age 1” and “Cor age 2” are the correlation between the behaviors in the first and second age classes considered, respectively. Not all studies reported non-significant correlations in which case the correlation is denoted as “NS”. Statistically significant non-zero correlations, as judged by the authors, are indicated by printing them in boldface. Under ‘Change’ we verbally describe the quantitative pattern of changes in correlations between the behaviors over age classes. Under ‘Ref’ a number is given referring to the study as cited in the main text.
